# Inefficient Preparatory fMRI-BOLD Network Activations Predict Working Memory Dysfunctions in Patients with Schizophrenia

**DOI:** 10.3389/fpsyt.2016.00029

**Published:** 2016-03-18

**Authors:** Anja Baenninger, Laura Diaz Hernandez, Kathryn Rieger, Judith M. Ford, Mara Kottlow, Thomas Koenig

**Affiliations:** ^1^Translational Research Center, University Hospital of Psychiatry and Psychotherapy, University of Bern, Bern, Switzerland; ^2^San Francisco VA Medical Center, San Francisco, CA, USA; ^3^Center for Cognition, Learning and Memory, University of Bern, Bern, Switzerland; ^4^Department of Psychiatry, University of California San Francisco, San Francisco, CA, USA

**Keywords:** schizophrenia, working memory, temporally coherent networks, state-dependent information processing, simultaneous EEG-fMRI, covariance mapping

## Abstract

Patients with schizophrenia show abnormal dynamics and structure of temporally ­coherent networks (TCNs) assessed using fMRI, which undergo adaptive shifts in preparation for a cognitively demanding task. During working memory (WM) tasks, patients with schizophrenia show persistent deficits in TCNs as well as EEG indices of WM. Studying their temporal relationship during WM tasks might provide novel insights into WM performance deficits seen in schizophrenia. Simultaneous EEG-fMRI data were acquired during the performance of a verbal Sternberg WM task with two load levels (load 2 and load 5) in 17 patients with schizophrenia and 17 matched healthy controls. Using covariance mapping, we investigated the relationship of the activity in the TCNs before the memoranda were encoded and EEG spectral power during the retention interval. We assessed four TCNs – default mode network (DMN), dorsal attention network (dAN), left and right working memory networks (WMNs) – and three EEG bands – theta, alpha, and beta. In healthy controls, there was a load-dependent inverse relation between DMN and frontal midline theta power and an anti-correlation between DMN and dAN. Both effects were not significantly detectable in patients. In addition, healthy controls showed a left-lateralized load-dependent recruitment of the WMNs. Activation of the WMNs was bilateral in patients, suggesting more resources were recruited for successful performance on the WM task. Our findings support the notion of schizophrenia patients showing deviations in their neurophysiological responses before the retention of relevant information in a verbal WM task. Thus, treatment strategies as neurofeedback ­targeting prestates could be beneficial as task performance relies on the preparatory state of the brain.

## Introduction

Deficits in working memory (WM) – defined as a system for temporary storage and manipulation of visual and phonological information (1) – represent a core feature in schizophrenia (2–5). There are studies discussing cognitive deficits in schizophrenia patients being mainly found in one specific domain, as for example in verbal episodic memory (6). The most consistent finding across studies, however, seems to be a generalized impairment across neuropsychological measures including verbal and spatial WM tests (5, 7, 8). These WM deficits are highly treatment resistant (9) and are indirectly related to poor functional outcome (10, 11). Although earlier fMRI studies reported on the dorsolateral prefrontal cortex (dlPFC) as the critical brain structure contributing to faulty WM in schizophrenia (12–16), later studies found that larger functional networks were recruited for successful performance (17–19).

The concept of fMRI-BOLD temporally coherent networks (TCNs) refers to a set of brain regions being temporally coactivated and describes both resting-state and task-related networks (20). Both resting-state networks (21–25) and task-related networks (18, 19, 26, 27) are affected by schizophrenia, as revealed by connectivity between or within the networks, their activation strength, or their spatial and temporal characteristics. Alterations in the most studied TCN, the default mode network (DMN), were related to severity of positive (26) and both positive and negative symptoms (22) during an oddball task and resting state, respectively. During WM tasks, the amount of DMN deactivation was linearly related to task demands (28), a balance that is behaviorally relevant in healthy subjects (29, 30). A disruption of the balance between DMN and task demands has been reported in patients with schizophrenia (31, 32), youth at high-risk for psychosis and early psychosis (33) as well as in unaffected siblings (34). According to the theory of state-dependent information processing (35), the brain’s state before a memory trial begins affects the subsequent neuronal response to internal or external stimuli such that greater DMN activation before stimulus presentation was linked to more errors in a flanker task (36) as well as in a stop signal task (37).

Measuring EEG and fMRI simultaneously has become an established tool for basic as well as clinical research since the first pilot study proved its feasibility (38). With the combination of these complementary methods, well-localized hemodynamic activity from fMRI can be related to neural activity from the EEG (39). A combined EEG-fMRI study with healthy subjects revealed the DMN being negatively correlated with frontal theta power in a resting condition (40), consistent with enhanced frontal EEG theta power in tasks requiring WM and focused attention (41–44). The same inverse association between frontal theta power and DMN was observed during the retention period of a verbal Sternberg WM task (45). This was replicated and extended in a recent study using the same WM paradigm within the framework of state dependency: four prestimulus fMRI-BOLD TCNs modulated three EEG frequencies during the subsequent retention interval when memoranda had to be held in WM (46). Importantly, their analysis provided temporal information about the relationship: prestimulus DMN activity modulated poststimulus frontal theta power. There is evidence from EEG studies during WM tasks that in schizophrenia, frontal midline (FM) theta power is reduced (47, 48), but no study has investigated the temporal relationship between DMN activity and neural activity during retention as reflected in FM theta.

The role of EEG alpha power linked to WM is somewhat controversial with reports of both load-dependent increases and decreases at different scalp sites from posterior, central to parietal areas (44, 46, 49–51). Greater WM loads are related to increased EEG power in the beta band at occipital (45, 46) and temporal sites (52, 53).

During rest, several TCNs are linked to specific EEG frequencies. A study of Jann and colleagues concluded that TCNs representing higher cognitive functions including self-referential, attention, and memory processes as the DMN and left and right working memory networks (WMNs) among others were positively related to higher frequencies (alpha and beta band) and had unique topographic frequency distributions (54). Applying the same method, a shift of the EEG spectral correlates of TCN fluctuations toward lower frequencies was detected for the DMN (from beta toward theta and alpha band) and the left WMN (from alpha and beta toward theta band) in schizophrenia patients (55). This finding further supports the notion that specific topographical associations of TCNs with frequencies are functionally relevant and aberrant in this patient population.

The goals of this study were to further extend previous results of Kottlow et al. (46) to schizophrenia. Exploring WM in a state-dependent manner may yield novel insights into deviations of cognitive functioning in this severe mental illness. Therefore, we investigated the effect of four prestimulus TCNs – the DMN, the dorsal attention network (dAN), the left and right WMNs – upon three EEG frequency bands (theta, alpha, and beta) during WM retention in patients compared to controls. First of all, we aimed at replicating findings in healthy controls to the previous study. Second and more importantly, we wanted to know if we could find evidence for a putative link between the well-known resting-state abnormalities, as seen both in spectral EEG deviations [e.g., Ref. (56)] and differences in fMRI-BOLD TCN dynamics [e.g., Ref. (32)], and the task-induced activation of WM-related brain functions, measured through changes in FM theta. In other words, we wanted to know if it is possible to find support for the hypothesis that a dysfunctional activation of WM functions in schizophrenia may follow from abnormalities in prestimulus resting-state activity. Thus, we expected the previously reported and WM relevant inverse association between the DMN and FM theta band to be reduced in patients.

## Materials and Methods

The preprocessing and analysis methods are identical to those used by Kottlow et al. (46). As the output data are multidimensional including the different TCNs, frequency bands, number of electrodes, load levels, as well as the two groups, we had to deal with the problem of multiple testing. By means of two analysis methods that eliminate the problem of multiple testing across electrodes, namely, the topographic consistency test (TCT) and the topographic analysis of variance (TANOVA), we reduced this problem (see Relative EEG Load Effects for further details). In addition, the findings of the mentioned paper were used to *a priori* limit the number of hypotheses that we considered.

### Subjects

We included 17 patients (14 males, 3 females; mean age: 34.62 ± SD: 8.79 years) and 17 age and gender matched healthy control subjects (14 males, 3 females; mean age: 31.62 ± SD: 7.06 years) in the study. Data from nine of these controls were included in Kottlow et al. Healthy control subjects were recruited *via* word of mouth.

All subjects satisfied standard inclusion criteria for participation in MRI studies, were right-handed, and had normal or corrected-to-normal vision. All refrained from caffeine and nicotine at least 4 h and alcohol 14 h prior to the experiment.

Patients were recruited at the University Hospital of Psychiatry in Bern, Switzerland. They were diagnosed according to the ICD-10 and DSM-IV either with schizophrenia (F20.0–F20.3; 295.1–295.4/295.6; *N* = 9) or acute and transient psychotic disorder (F23.0–F23.2; 297.1/298.8; *N* = 8). Patients medicated with the atypical antipsychotic medication clozapine were excluded due to its adverse effects on the EEG (57). Other exclusion criteria included comorbidities for other psychiatric disorders, substance abuse (except for nicotine and caffeine), and neurological or other severe medical conditions. Sixteen of the 17 patients received antipsychotics (typical = 1; atypical = 15), 1 received antidepressants, 2 received mood stabilizers, and 2 received tranquilizers. Healthy controls had no history of psychiatric and neurologic disorders and abuse or dependence on psychoactive medication or drugs other than nicotine and caffeine.

Study procedures were approved by the local ethics committee of the canton of Bern, Switzerland (KEK no. 192/05). All subjects gave their written informed consent prior to examination and were aware that they could drop out at any time point and without cause and for patients without treatment consequences. Patients indicating poor understanding of the study were excluded. Treating psychiatrists confirmed the patient’s ability to give informed consent. Participation was unpaid. The characteristics of the subjects and clinical variables are given in Table 1.

**Table 1:** **Descriptive and clinical variables of subjects**.

	Patients (*N* = 17)		Controls (*N* = 17)		*T*-tests
	(m/f)	%	(m/f)	%	
Gender	14/3	82.4/17.6	14/3	82.4/17.6	

	**Mean**	**SD**	**Mean**	**SD**	***p* (df = 32)**

Age (years)	34.62	8.79	31.62	7.09	0.281
WAIS III (*t*-values)	43.47	7.12	55.88	5.18	<0.001
Duration of illness (months)	66.66	67.86			
Number of episodes	3.88	3.44			
CPZE	344.17	236.28			
DPZE	0.2	0.7			
PANSS positive	12.65	4.47			
PANSS negative	12.82	7.76			
PANSS total	52.12	22.94			

*WAIS III, Wechsler adult intelligence scale (four subtests: similarities, digit span, the block design test, and the digit symbol-coding test); CPZE, chlorpromazine equivalence dosage; DPZE, diazepam equivalence dosage; PANSS, positive and negative syndrome scale*.

### Task and Procedures

The study was conducted on 2 or 3 separate days. On the first day, the neurophysiological measurements were held between 8 a.m. and 12 a.m. at the Inselspital of Bern, Switzerland. Within a week, cognitive performance was assessed by means of four subtests of the Wechsler adult intelligence scale (WAIS III; similarities, digit span, the block design test, and the digit symbol-coding test). Additionally, a clinical-diagnostic interview was performed with patients during the same day as the cognitive assessment or on a third day close to the other assessment days.

The procedures for the neurophysiological measurements were as follows: first, the EEG cap was placed followed by 8 min resting-state acquisition consisting of alternating blocks of 2 min eyes open and closed outside of the scanner. Before going into the MR scanner, subjects performed a short practice version of the WM task. Subjects were placed carefully in the scanner for the simultaneous EEG-fMRI measurements consisting of another 8 min resting state and then the performance of the WM task. After removal of the EEG cap, the anatomical sequences were executed.

In the scanner, stimuli were presented *via* goggles (VisualStimDigital MR-compatible video goggles; Resonance Technology Inc., Northridge, CA, USA). The visual angle of the stimuli was 60° with a resolution of 800 × 600 pixels and 60 Hz refresh rate. An in-house fabricated MR compatible response box was used. Stimuli were delivered and responses registered using E-Prime (Version 2.0.10.553, Psychology Software Tools, Inc.).

**Figure 1:**
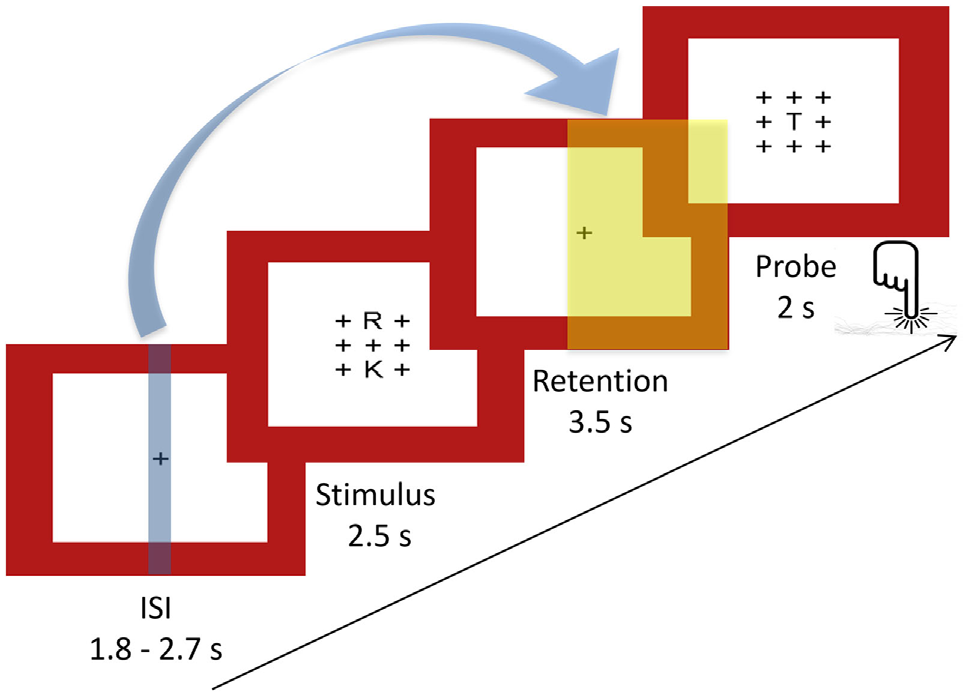
**Sternberg WM task: experimental design of a single trial**. Indicated with the blue arrow and the blue and yellow plains are the time points of fMRI (blue) and EEG (yellow) markers extracted for the covariance mapping (ISI, inter-stimulus interval, mean: 2 s).

**Figure 2:**
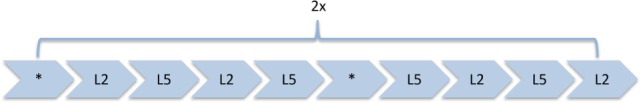
**Sternberg WM task: task design**. Four blocks of each load (load 2 = L2, load 5 = L5) alternating among baseline conditions (*). Each block contains four trials per load and was followed by a fixation of 2.5 s added to the ISI (+).

To assess verbal WM processes, a version of the Sternberg Item Recognition Paradigm [SIRP; Ref. (58)] adapted from Michels et al. (45) was used. This paradigm allows the temporal separation of the encoding of memory items (*encoding phase*), retaining them (*retention period*), and their retrieval (*probe period*). Either two (*load 2*) or five (*load 5*) consonants were presented in an array of 3 × 3 items with the remaining positions being plus signs (+). The positions of the consonants were counterbalanced across trials, with the center one being excluded. This array was presented in black font on a white background surrounded by a red frame. One trial included a stimulus array presented for 2.5 s, followed by the retention period consisting of a centered fixation cross (+) for 3.5 s. Then, in the probe period, one consonant was presented in the center for 2 s. There was a variable duration (range: 1.8–2.5 s, mean: 2 s) inter-stimulus interval (ISI) before the next trial with the centered fixation cross. Subjects had to indicate by bottom press whether the probe letter was presented before or not (the use of the right index and middle finger to indicate “yes” or “no” answers was counterbalanced between subjects). The task included 8 blocks per load condition comprising 4 trials each, resulting in 32 trials per load condition. Between blocks, the fixation cross was displayed for 2.5 s. Five times throughout the task, a centered fixation star (*) was projected during 24.5 s of rest. Task duration was 13 min. Figure 1 illustrates the experimental design for one trial, and Figure 2 the task design.

### EEG Acquisition and Preprocessing

The EEG was measured using a 96-channel MR compatible system from Brain Products (Gilching, Germany; Input range: 16.3 mV, resolution: 16 bit). Ninety-two electrodes were mounted in an elastic cap according to the international 10–10 system. Additionally, two channels each were used to measure the electrocardiogram (ECG; below the clavicles) and the electrooculogram (EOG; below the eyes). Electrode Fz was used as recording reference. Three BrainAmp MR compatible amplifiers were connected to 32 channels each and connected to the acquisition computer *via* fiber cables for safety reasons. The EEG was online bandpass filtered between 0.1 and 250 Hz and sampled with 5 kHz. We aimed at keeping electrode impedances below 20 kΩ while restricting the duration of entire EEG preparation to 1 h in order to avoid tiring the subjects. Across subjects, 90% of all electrodes had impedances below 25 kΩ, 5% were higher than 30 kΩ of which only 14.7% of subjects shared common electrodes and mean impedance was 17.5 kΩ. To avoid aliasing artifacts, the clock of the recording computer was synchronized to the clock of the MR system (10 kHz refresh rate), and each MR scan volume was automatically marked in the EEG data.

Preprocessing was performed using Vision Analyzer (Version 2.0.4.368; Brain Products, Gilching, Germany). Methods used for artifact removal are in accordance with previously published papers (46, 54, 55, 59) and briefly summarized in the following section. The EEG was corrected for artifacts including scan-pulse and cardio-ballistic artifact, using average artifact subtraction with a sliding window (60). Thereafter, for each subject, EEG files from outside and inside of the scanner were down-sampled to 500 Hz and concatenated. The resulting file was then bandpass filtered (1–49 Hz and a notch filter) and bad channels were disabled (in controls: one subject had four disabled channels, one subject had two, two had one channel; in patients: one had five disabled channels, five had one channel). Using an ICA-based approach, the EEG was further cleaned from remaining cardio-ballistic, scan-pulse, and eye movement artifacts. Components loading for artifacts were identified using visual inspection of their temporal dynamics, topographic maps, and the comparison of their power spectra inside versus outside of the scanner. The EEG of each subject was reconstructed from the remaining factors, and epochs containing residual scanner or movement artifacts were marked by visual inspection thereafter. Disabled channels were interpolated using a spherical spline interpolation. Furthermore, the ECG and EOG channels were removed, and the EEG was recalculated to average reference. Finally, the file was separated again into the resting state and verbal WM task.

### MRI Data Acquisition and Preprocessing

The recordings were performed in a 3-T Siemens Magnetom Trio MR Scanner (Siemens, Erlangen, Germany) with a 12-channel head coil. The functional T2*-weighted MR images were acquired with an echo planar imaging (EPI) sequence. The characteristics of the sequence were 250 and 406 volumes for the resting state and the Sternberg WM task, respectively, 35 slices, 3 mm × 3 mm × 3 mm, matrix size 64 × 64, FOV 192 mm × 192 mm, TR/TE = 1960 ms/30 ms.

The structural T1-weighted sequence (ADNI) had following parameters: 176 sagittal slices, slice thickness 1.0 mm, voxel size 1 mm × 1 mm × 1 mm, FOV 256 mm × 256 mm, TR/TE = 2300 ms/2.98 ms.

Preprocessing of the functional MRI data was done in SPM8 (Welcome Department of Imaging Neuroscience, London).^1^ First, slice time correction was performed, and the data were motion corrected to the mean image. Then, the anatomical T1 was coregistered to the mean image, followed by its segmentation into six tissue probability maps. Finally, the data were normalized and smoothed using an FWHM kernel of 6 mm × 6 mm × 6 mm.

### TCN Extraction

To obtain the temporal dynamics of the TCNs from our fMRI data, we applied a spatial–temporal regression implemented in the GIFT toolbox (61).^2^ As templates to reconstruct single subject’s components, we applied the four components of interest (DMN, dAN, left and right WMNs) from the group-ICA on 24 healthy subjects from the previous study (46). First, the fMRI-BOLD time series of each subject were preprocessed applying the variance normalization option of the toolbox for comparability reasons. Then, the four components of interest were back projected onto the subject’s time series. The resulting time courses were *z*-transformed and represent the percent signal strength of each TCN at each volume acquired over whole WM task duration (406 volumes total) of each subject.

### Evolution of TCNs over Average Task Trials

Based on previous findings, we expected the DMN and dAN to show opposing dynamics in healthy controls and that these dynamics would be relevant for performance (46, 62–65). To reveal whether differences in performance between patients and healthy controls could be related to the relative pre- or poststimulus network activations of the DMN and dAN, their mean dynamics where extracted from the time courses of these TCNs of the prestimulus period (−4.3 until −2.5 s before retention onset) and the retention period (0 until 3.5 s). These mean dynamics were then compared for each time point with a three-factorial ANOVA (2 × 2 × 2) with the factors network, load, and group. To check whether pre- to postdifferences could be ascribed only to the higher load condition (load 5), another ANOVA was performed including the factors pre–post, network and group for load 5 only (2 × 2 × 2 factorial design). Furthermore, separate ANOVAs were conducted for the prestimulus and retention periods for each TCN for the factors of load and group (2 × 4 = 8 ANOVAs with a 2 × 2 factorial design). Using in-house Matlab scripts, we additionally plotted the TCN dynamics. Therefore, in a time window from −4.3 to 5.5 in reference to the retention period each TCN time course was interpolated on a 0.1-s time scale using the Matlab spline interpolation and averaged across trials for each group and load condition. The five baseline periods were disregarded from that time window. Using *t*-test statistics, these averaged dynamics were compared every 0.1 s against 0 within (two-tailed one-sample *t*-tests) and between (two-tailed unpaired samples *t*-tests) load levels and groups.

### Spectral Power Differences during Resting State

To check for spectral power differences during the resting-state condition with eyes closed, the cleaned resting-state EEG file was segmented into the eyes closed condition only, resulting in 4 min of continuous EEG. Then, the EEG was segmented into equally sized segments (2.048 s) and a fast Fourier transformation (resolution: 0.48828 Hz, hanning window: 10%) was performed. Afterwards, frequency bins were collapsed into the three frequency bands theta (3–7 Hz), alpha (8–12 Hz), and beta (13–20 Hz). For each subject, the average across all segments and the global spectral power across all channels were calculated. Finally, patients and controls were compared with frequency bin wise *t*-tests.

### Relative EEG Load Effects

Similar to Kottlow et al. (46), relative load effects defined as ratio of high versus low load were calculated. Therefore, the cleaned EEG data were segmented from 1 to 3.5 s within the retention period for correctly answered trials for each load separately. For each frequency band (theta, alpha, and beta), relative load effects were computed. Furthermore, the software package Ragu,^3^ which is based on randomization statistics was used to check the spatial stability of the load effects for each frequency across subjects per group with the TCT [for a detailed description of the methods, see Ref. (66)]. As this test is run across all electrodes at once, the problem of multiple testing is being reduced. Where significant TCTs resulted for the same frequency bands per group, topographic analyses of variance (TANOVAs) were run in Ragu to check for significant topographical differences between groups. With this analysis too, the problem of multiple tests is decreased due to the comparison of topographies as a whole, not single electrodes. Other comparisons are not meaningful due to the lack of consistency across subjects.

### Covariance Mapping

As the aim of the study was to explore state dependency within a WM task incorporating both fMRI and EEG measures at different time points, we used a method suitable for multivariate datasets, the so-called covariance mapping. Hereby, EEG scalp topographies representing the channel-wise covariance of a single EEG parameter (such as power at a specific frequency) with another continuous external variable (such as reaction times, but also a single fMRI parameter) are calculated [for further details, see Ref. (67)]. Positive covariance means both variables fluctuate in the same direction together whereas negative values point toward an inverse relationship between the two. We here examined the lagged coupling of the relative signal strengths from four fMRI TCNs at prestimulus with the amplitude of three EEG frequency bands during the retention period of a WM task. Therefore, the cleaned EEG data of the WM task were divided into segments containing the last 2.5 s of the WM retention period for correctly answered trials separately for each load condition and each subject. To extract spectral amplitude of frequencies, a continuous complex Gabor transformation spanning frequencies from 2 to 20 Hz with an envelope having its half maximum at the latency of a full cycle of the oscillation was applied. The single trial epochs were pooled into the previously used frequency bands theta (3–7 Hz), alpha (8–12 Hz), and beta (13–20 Hz). Covariance maps were calculated relating the level of every TCN before stimulus presentation (−3.5 s before retention onset) with the EEG frequency band data (the last 2.5 s within the retention period) for both load levels and every subject separately. Thus, 24 covariance maps were obtained per subject (4 TCN × 3 Frequency bands × 2 loads).

The subsequent analyses were performed again with the software package Ragu. First, TCTs were run on the covariance maps averaged over the whole time window (1.0–3.5 s within the retention period) for each TCN and frequency band across subjects per group. We further explored differences in covariance maps between groups if both groups showed significant TCTs for the same conditions according to the procedure for the relative load effects. For covariance maps that were significantly consistent within both groups, TANOVAs were computed to check whether the topographies of the covariance maps were significantly different between groups and load levels. Significant effects were then visualized using *t*-maps. To explore whether covariance maps were affected by medication (chlorpromazine equivalence dosage = CPZE), the severity of symptoms (PANSS positive, negative, and total scores), or cognitive performance (*t*-values of summed WAIS III subtests), for every TCN, we ran TANCOVAs with each of these variables as covariates.

Finally, based on the existing literature, we expected that control subjects would show a focal inverse relationship of theta at frontal electrodes [Fz: Ref. (40, 45) or AFz: Ref. (46)] with DMN activation in the prestimulus interval (46) selectively for the high-load condition. We therefore tested in a FM theta analysis the covariance of these specific electrodes (Fz and AFz) for load and group differences.

**Figure 3:**
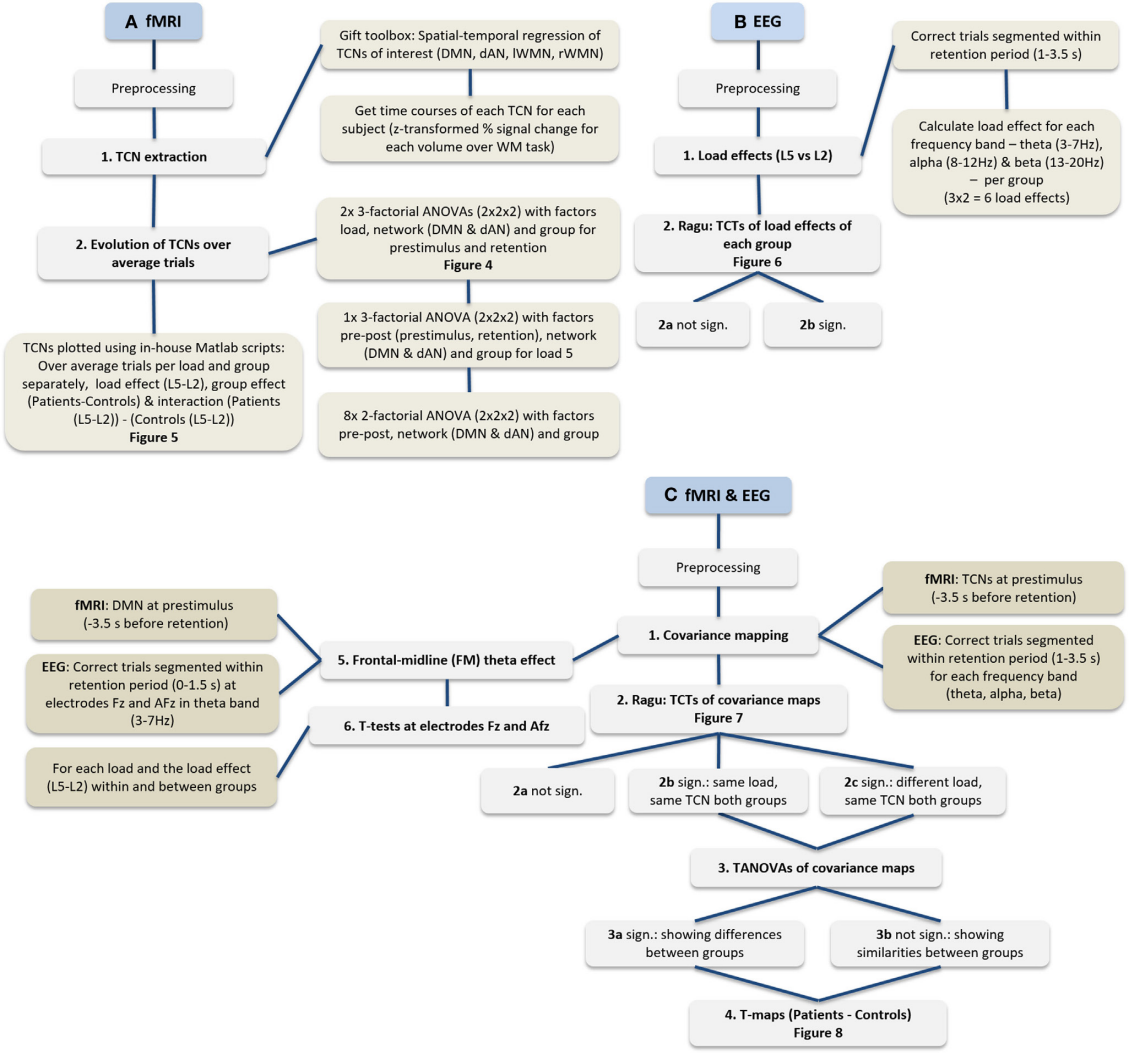
**Flowchart of performed analyses**. **(A)** Procedure of fMRI data only, **(B)** EEG data only, and **(C)** the procedure integrating fMRI and EEG.

Figure 3 provides an overview of all analyses steps applied on the EEG and fMRI data separately as well as their joint analyses.

## Results

### Behavioral Data

For an overview of the results see Table 2. We performed two ANOVAs for reaction times and accuracies (2 × 2 factorial design with factors load and group). The ANOVA for reaction times resulted in a significant main effect load (*p* ≤ 0.001) and group (*p* = 0.023), but no significant interaction effect (*p* = 0.708). The ANOVA run for accuracies (percent correctly answered trials) yielded a significant main effect load (*p* = 0.001) and interaction of load by group (*p* = 0.017), but scarcely no significant main effect group (*p* = 0.056). To further explore this result, independent sampled *t*-tests were run to check for differences in accuracy between groups for each load level separately. It resulted that the significant interaction from the ANOVA was driven by a significant effect of load 5 only (load 2: *t* = 0.585, df = 32, *p* = 0.563; load 5: *t* = 2.588, df = 32, *p* = 0.014). The performance across both loads ranged in controls from 81.25 to 100% and in patients from 62.5 to 100%, indicating that all subjects were able to perform the task above chance level.

**Table 2:** **Behavioral results of the WM task**.

	Patients (*N* = 17; 14 = m, 3 = f)		Controls (*N* = 17; 14 = m, 3 = f)	
	Mean	SD	Mean	SD
RT all (ms)	1162.16	186.48	975.98	224.20
RT L2 (ms)	1018.7	196.4	852.07	205.28
RT L5 (ms)	1283.25	225.62	1099.02	267.03
Acc all (%)	89.43	9.6	94.76	4.97
Acc L2 (%)	94.12	8.26	95.59	6.26
Acc L5 (%)	84.93	13.18	93.93	5.68

*Means and SDs of reaction times (RTs) and accuracies (Acc) for each load level (L2, L5) separately and merged (All) for each group*.

### Evolution of TCNs

**Figure 4:**
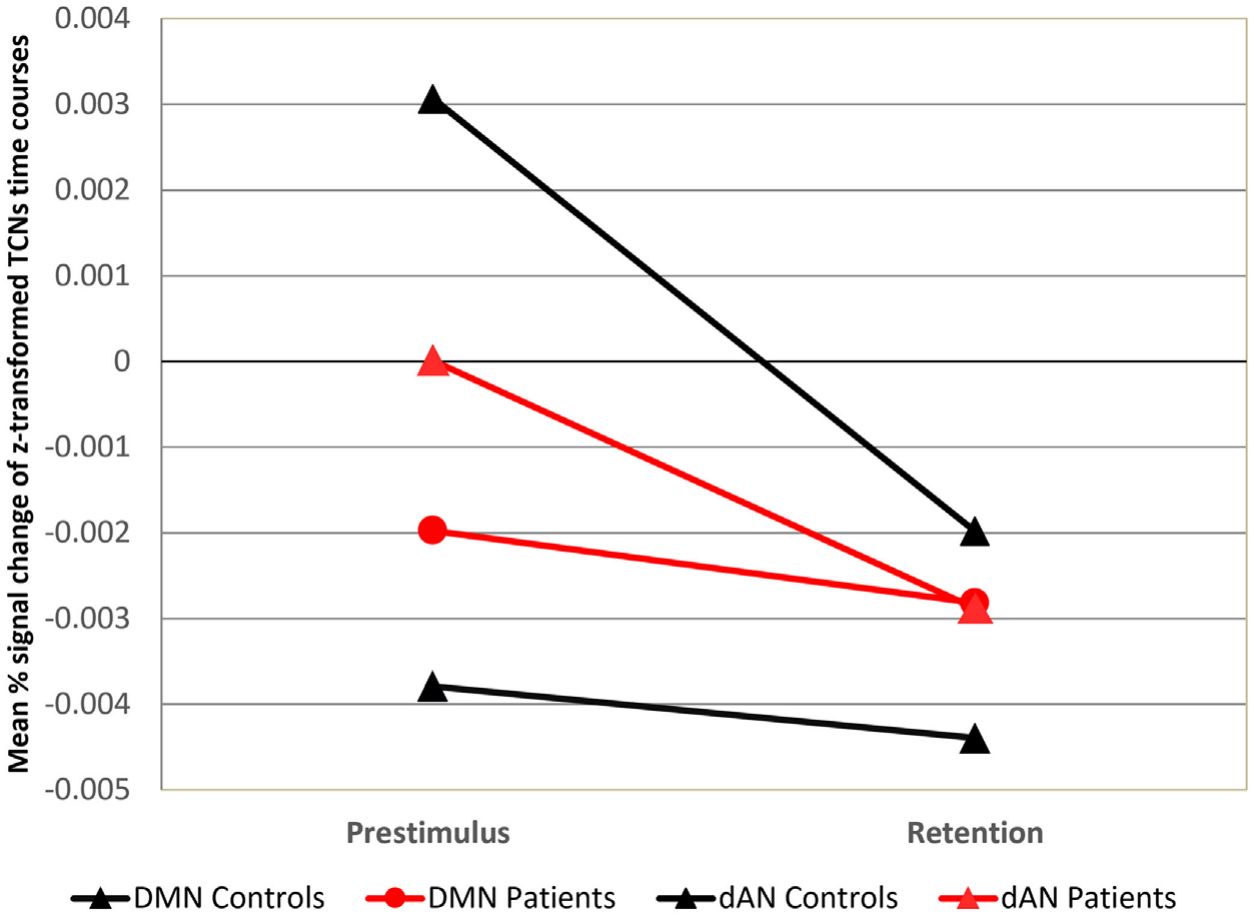
**Mean DMN and dAN from pre- to poststimulus in the WM task**. Mean DMN and dAN dynamics at prestimulus and retention intervals for each load and group. *X*-axis: time points (prestimulus and retention period), *Y*-axis: mean percent signal change of variance normalized, and *z*-transformed TCNs.

The mean activations of pre–post (prestimulus and retention period) for the DMN and dAN for both loads are shown in Figure 4. The three-factorial ANOVA for the prestimulus interval revealed a nearly significant interaction of TCN by group (*p* = 0.053), whereas no significant effect arose for the retention period. Further analysis indicated that healthy controls showed the anti-correlation of the two networks known in the literature (46, 62, 63, 68), the DMN was significantly lower than the overall mean level (one-sample *t*-test, *p* = 0.0058) as opposed to a significantly enhanced dAN (one-sample *t*-test, *p* = 0.029). In patients, no systematic deviations from the mean could be observed. In addition, significant performance differences were limited to load 5. The respective ANOVA (2 × 2 × 2 factorial design with factors network, pre–post and group for load 5) supported this finding by a significant interaction effect of the factors pre–post and group (*p* = 0.028). The ANOVAs run separately for each network at prestimulus and retention period with factors load and group revealed a significant main effect of load (*p* < 0.001) for the DMN and a main effect of group (*p* = 0.023) for the dAN at prestimulus period. In the retention period, a significant load effect (*p* = 0.013) and a group effect (*p* = 0.021) resulted for the WMN on the right.

**Figure 5:**
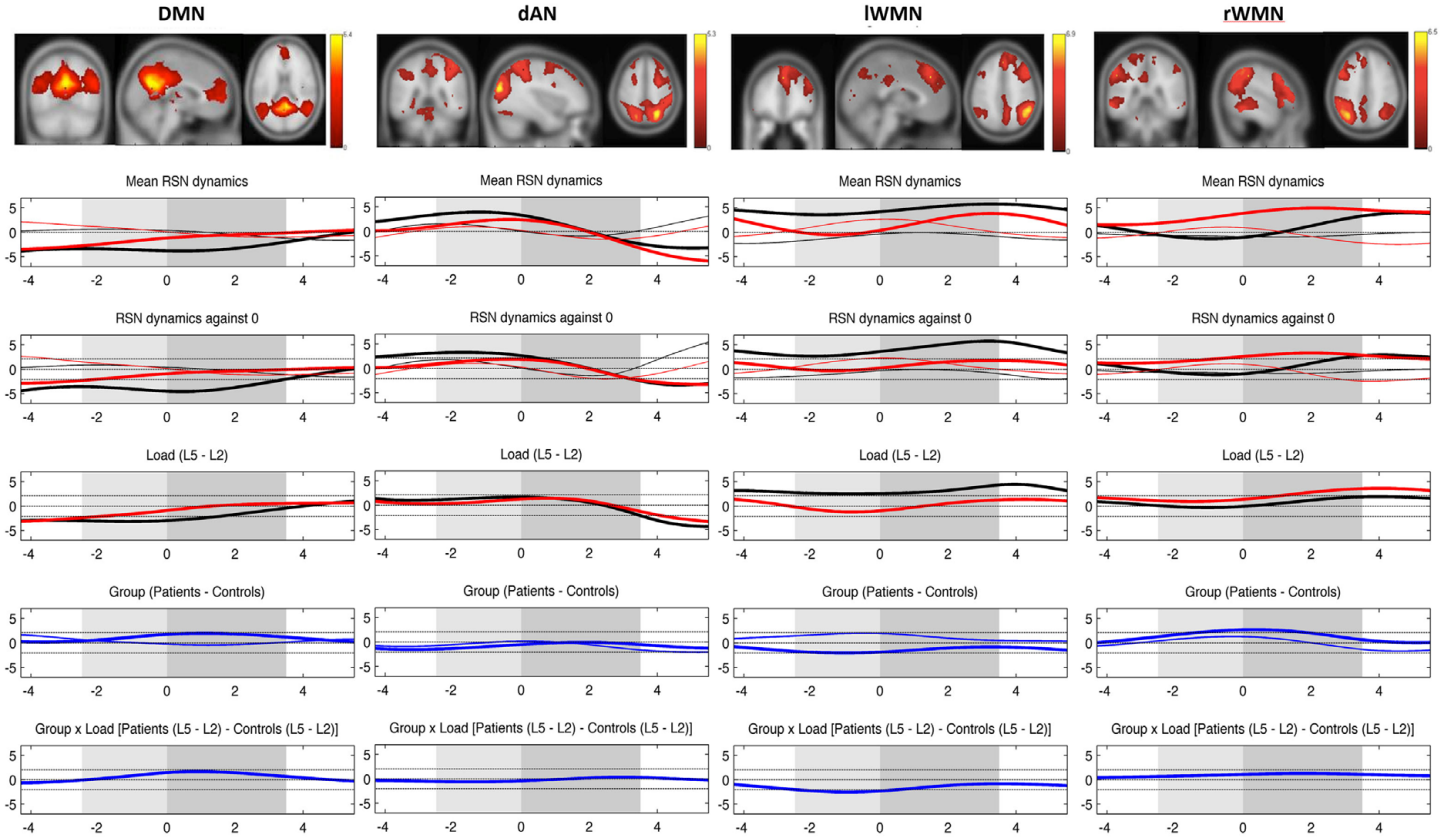
**Mean evolution of TCNs over average trials**. Upper plot shows the templates for each TCN from the study of Kottlow et al. with the three orthogonal slices through areas of maximum activation (only positive values; for detailed information about included regions, see Table S1 in Supplementary Material). Lower plots display the mean network evolutions over average trials for each load (thin line = load 2; thick line = load 5) and each group (black line = controls; red line = patients). Dashed lines indicate significance of the *t*-tests (two-sided, *p* = 0.05): (1) mean evolutions, (2) mean evolutions against 0, (3) load effect, (4) group effect (blue thin line = load 2; blue thick line = load 5), and (5) interaction of group by load. *X*-axis: time over trial [prestimulus: −4 to −2.5 s; stimulus (light gray block): −2.5 to 0 s; retention (dark gray block): 0–3.5 s; probe: 3.5–5.5 s], *Y*-axis: percent signal change of variance normalized, and *z*-transformed TCNs’ time courses.

The time resolved event-related TCN dynamics are represented in Figure 5. For healthy controls, the fluctuations of their four TCNs were comparable to those seen in the preceding study (46). Main findings were the significant load-dependent decrease of the DMN from prestimulus until the retention period, while the dAN displayed a different pattern with higher activation in the prestimulus period decreasing in the middle of the retention period thereafter. A stronger recruitment of the WMN on the left was present over the entire trial period in the more challenging condition. This was not the case for the WMN on the right, which was even suppressed during encoding of the memoranda and the first second of the retention period.

In patients, a reduced suppression of the DMN for trials at load 5 was seen. The group effect reached significance from the beginning until 2.2 s of the retention period (*p*-values: ≤0.048 and ≥0.031). The evolution of the dAN was comparable to the one in control subjects apart from a reduced activation level for higher load trials during prestimulus as well as for lower load trials in the probe period (significant group effect from 1.4 s until the end of the 2-s probe period, *p*-values: ≤0.049 and ≥0.048). Comparing the evolution of left and right WMNs, patients did not show the lateralization effect seen in controls for the more difficult WM condition: their WMN on the left was not as activated as in controls (in spite of showing the same evolution pattern), whereas the WMN on the right grew stronger toward the end of the stimulus and over the whole retention period (group effect with maximum *p*-value of .02). Finally, there was a group by load interaction in the left WMN during stimulus period (*p*-values: ≤0.045 and ≥0.0162), which could be explained by controls having greater left WMN recruitment at load 5 and smaller at load 2 than patients during encoding the memoranda.

### Spectral Power Differences during Resting State

Regarding the preprocessing of the EEG data *via* ICA, there was no significant difference in the number of removed components between groups (controls: mean = 20.5, SD = 2.7; patients: mean = 18.4, SD = 3.4 out of 64 components, *t* = 1.994, df = 32, *p* = 0.055).

The comparison of the power spectra between the groups resulted in a significant elevation of theta (4.7–6.6 Hz, *t* > 2.0369, *p* = 0.05, double-sided) as well as beta (15.1–16.5 and 18.1–20 Hz, *t* > 2.0369, *p* = 0.05, double-sided) band in patients compared to controls (Figure S1 in Supplementary Material).

### Relative EEG Load Effects

**Figure 6:**
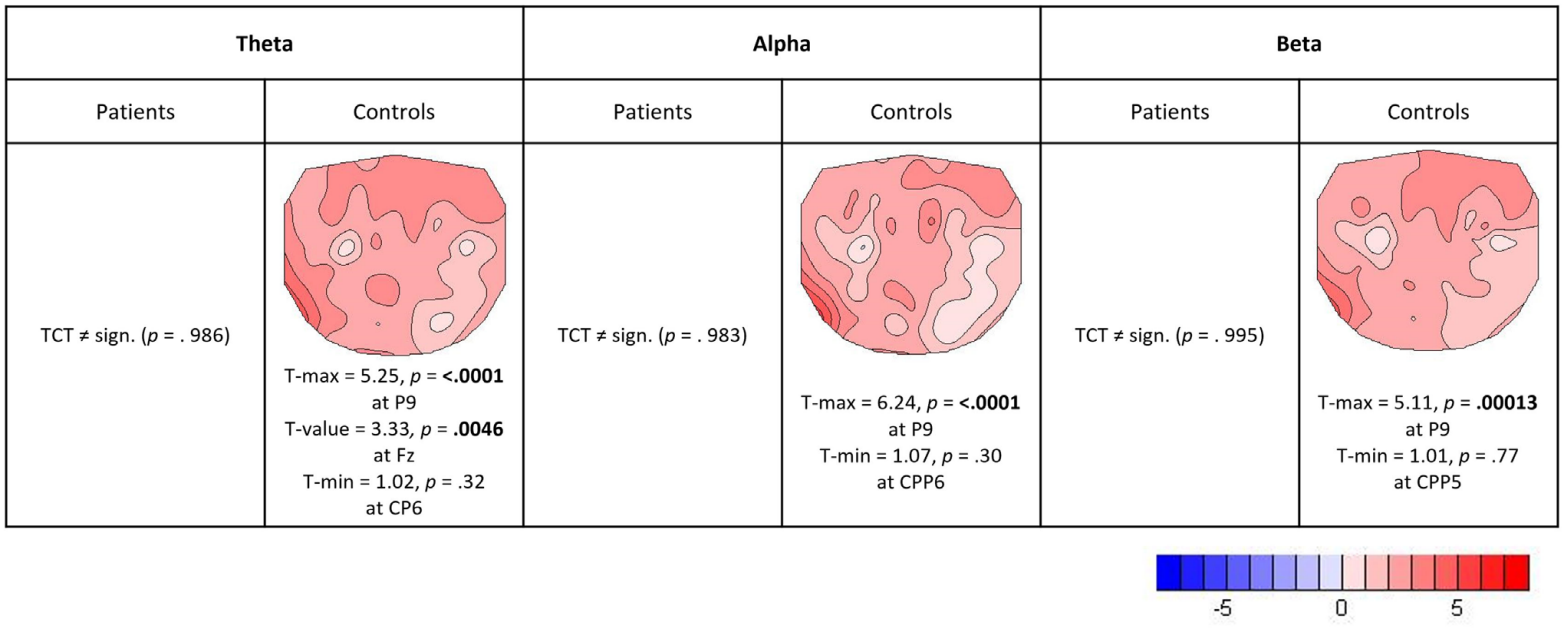
**Relative load effects (load 5 versus load 2)**. *T*-maps with maximum and minimum *t*-values and their significance level (two-sided, *p* = 0.05) are indicated for those frequency bands and groups where the TCT reached significance.

Topographic consistency tests were done in Ragu to check the stability of load effects for each frequency band and group (3 frequency bands × 2 groups = 6 TCTs). In controls, all three TCTs of their load effects revealed significantly consistent topographies (*p*-values: theta = 0.0002, alpha = 0.0002, and beta = 0.0002). In patients, however, none of the load effects had significant topographic consistency (*p*-values: theta = 0.986, alpha = 0.983, and beta = 0.995). Figure 6 shows that controls had positive load effects in all frequency bands that included frontal and left temporo-parietal sites and had a local maximum at the expected position Fz in the theta band (maximum *t*-value at P9 = 5.25, *t*-value at Fz = 3.33). No TANOVA could be performed, as there was no significant effect in patients to be compared to the topography of controls.

### Covariance Maps

**Figure 7:**
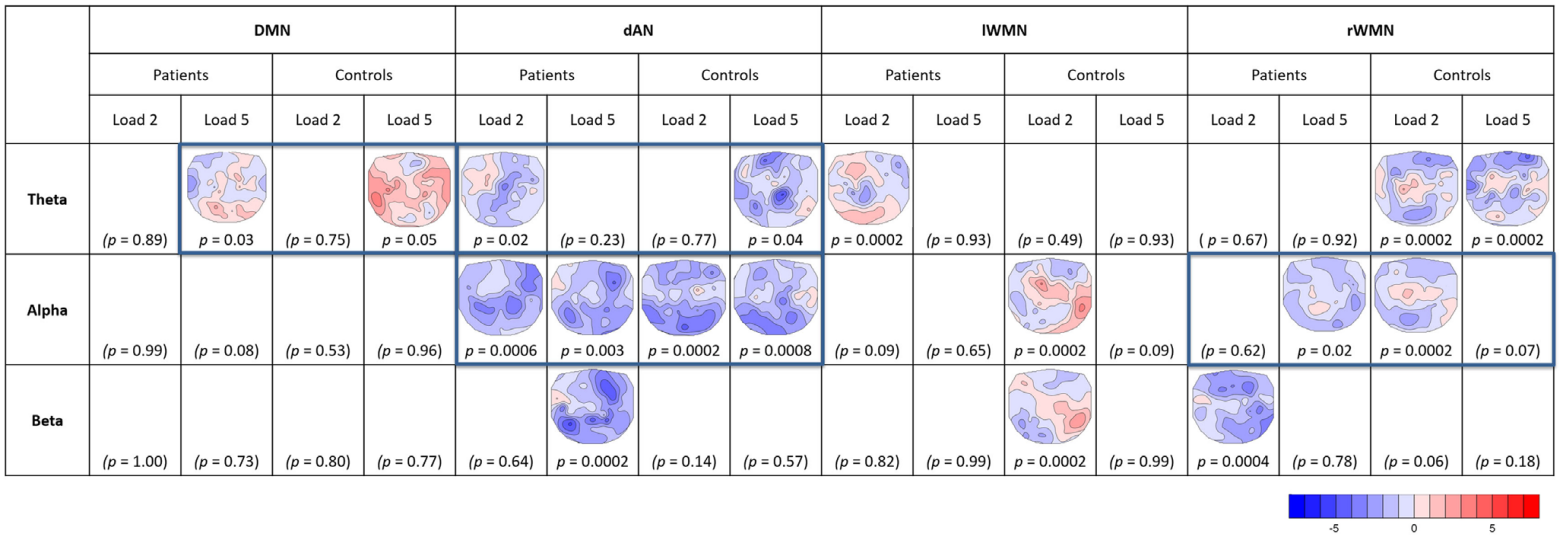
**Overview of significant TCTs of covariance maps**. For each TCN (DMN, dAN, left and right WMNs), frequency band (theta, alpha, and beta), and load (L2, L5) per group (patients, controls). *T*-maps of significant TCTs (two-sided, *p* = 0.05) are indicated (blue = negative, red = positive covariance). Framed maps were further compared between groups.

**Figure 8:**
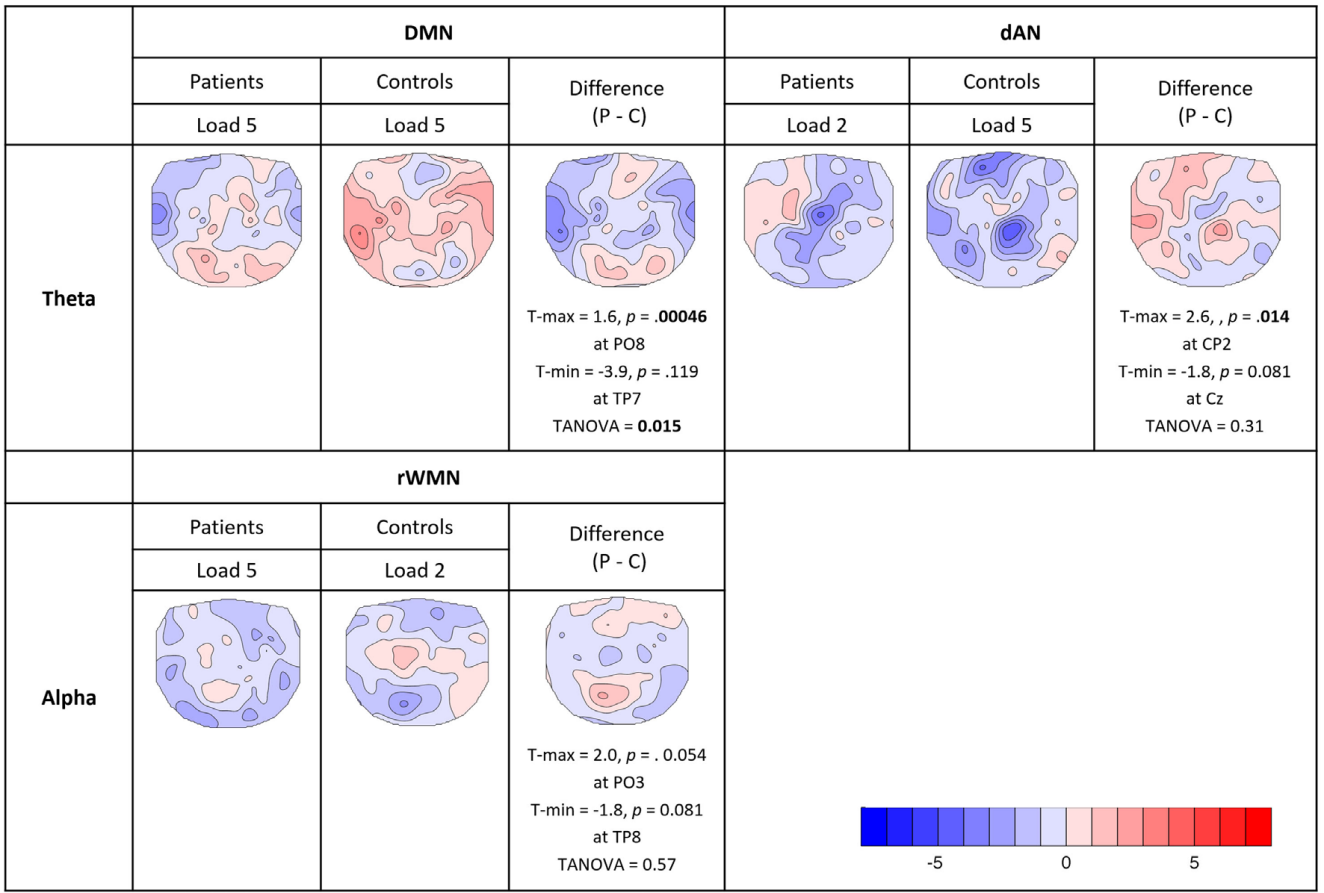
**Comparison of consistent covariance maps between groups**. Where the TCTs were significant for both groups within the same TCNs and frequency bands, TANOVAs were run to check for significant spatial differences or similarities. Displayed in the figure are *t*-maps for each group and their difference (patients − controls) showing electrodes with maximum and minimum *t*-values, the significance levels (two-sided, *p* = 0.05) and the results of the TANOVAs.

For the analysis of the covariance maps, the data of 16 patients and 16 matched controls were analyzed as 1 patient lacked enough clean EEG segments (16 good segments out of 64). Healthy controls showed a higher number of consistent covariance maps than patients over all TCNs and frequency bands (out of 24 total TCTs, 8 were significant in patients and 9 in controls, see Figure 7). Highlighted in the figure are these maps, which were compared further between groups (being referred to cases 2b and 2c in Figure 3C). Coupling between the DMN at prestimulus and theta frequency during retention in high-load trials was significantly different in the two groups representing case 3a in Figure 3C (TANOVA: *p* = 0.015; *t*-min = −3.9, *p* = 0.119 at TP7; *t*-max = 1.6, *p* = 0.00046 at PO8, see Figure 8). Patients showed in general a rather left lateralized and a much more extended inverse coupling than controls.

Looking at the dAN with theta band, patients’ covariance map of load 2 resembled controls map of load 5. Their topographies seemed visually similar with mainly negative covariance at frontal, central, and parietal electrodes, which was further confirmed by the TANOVA not yielding a significant difference between the groups (TANOVA: *p* = 0.31, case 3b in Figure 3C.). For the dAN with alpha frequency, both load conditions had significant TCTs for both groups and they visually all looked very similar having extended negative couplings matching again Kottlow et al. (see Figure 7, case 3b in Figure 3C). We assessed load, group, and group by load interactions with the TANOVA and found no significant effects, suggesting that there was no specific topographic coupling of the dAN with alpha for load or group. Finally, we found significant TCTs of the right WMN with alpha for patients at load 5 and controls at load 2. Similar to the dAN with theta, the TANOVA revealed no significant effects (TANOVA: *p* = 0.57, case 3b in Figure 3C), thus both covariance maps resembled each other. The maps demonstrated again mainly inverse but also some positive association at centro-parietal areas.

None of the computed TANCOVAs including covariates as medication, severity of symptoms, and cognitive performance was significant, and we therefore have no reason to believe that our covariance maps were influenced by any of these factors.

In controls, the planned FM theta analysis indicated the expected inverse relation of prestimulus DMN level with frontal theta specifically for the high-load condition. However, this effect was only significant in the mean of the first 1.5 s of the retention period. Namely, theta covariance during load 5 at Fz was more negative than 0 (*t* = −1.76, df = 15, *p* = 0.049, single-sided), and significantly more negative for the load 5 compared to the load 2 condition (*t* = −2.72, df = 15, *p* = 0.016). In patients, no consistent covariance was found during load 5 (*t* = 0.22, df = 15, *p* = 0.83), and no difference between loads was found (*t* = 0.73, df = 15, *p* = 0.48). The load-dependent effects in FM theta covariance (load 5 − load 2) were significantly different between groups (*t* = 2.25, df = 30, *p* = 0.032). At electrode AFz, these effects were far weaker and not significant. We note that while in the mean, a negative covariance of prestimulus DMN activation and FM theta during retention was present over the entire analysis period, the effect was only significant in a time window before the analysis period we had initially chosen. Since this analysis period was primarily chosen based on previous literature that had not considered such pre- to poststimulus interactions, we reanalyzed the data of the study by Kottlow et al. (46) that first reported this type of interactions. The reanalysis of this data showed that the described effect could also be found in the early time period as reported here. We therefore felt it was more appropriate to adjust the analysis window rather than rejecting the result due to the difference in timing.

## Discussion

To expand the current understanding of the neurophysiology underlying WM deficits in schizophrenia, we explored the modulatory effect of prestimulus fMRI-BOLD networks on EEG spectral power during the subsequent memory retention interval.

Overall, we found consistent and specific topographies of the relative EEG load effects as well as a coupling of prestimulus TCNs with EEG oscillatory frequencies during the retention period. We replicated earlier findings reported by Kottlow et al. (46). Namely, healthy subjects showed similar dynamics of the TCNs, and we mainly saw load effects pointing toward significant changes for more difficult trials. Thus, we can argue that the WM task of load 2 recruits few cognitive resources resulting in little change captured by the neurophysiological measures. Consequently, the prestimulus neurophysiological state of the brain relates to subsequent processing of information in a demand-dependent manner and may therefore support or interfere with cognitive functioning.

Regarding the patient data, our resting-state analysis replicated established reports on increased power in slow frequencies as theta and also higher frequency beta band (55, 56, 69–72).

In general, subjects were significantly slower to respond to more difficult trials. In agreement with existing literature, patients had significantly prolonged reaction times for the execution of the WM task (14, 31, 33). Interestingly, there is a recent publication that could link reaction time to prestimulus theta band activity in an identical WM task in healthy subjects (73).

The accuracy level significantly dropped for the higher load level over all subjects and patients were significantly less accurate in responding to load 5 trials compared to control subjects (84 versus 94%). However, the overall high behavioral accuracy (mean patients: 89.43%, mean controls: 94.76%) indicated that the higher load condition was still within the range of WM capacities in patients. According to studies including even higher load conditions in a similar WM paradigm (18, 33, 74), we would expect that just a small increment of the difficulty level would have led to further substantial drops in patients’ performance.

Our results in controls also demonstrated the known theta band load effects during the retention period and deviations in the patient data. In patients, no evidence for consistent load effects in any of the three investigated frequency bands was found. The lack of the classical theta band load effect in patients with schizophrenia is consistent with WM dysfunction reported in the literature. That alpha band was not affected by load in patients and may reflect counter-veiling roles of alpha oscillations to inhibit irrelevant brain regions while supporting demands on attention (75). Finally, beta band power seems to be less affected by load than alpha and theta. The increase in left parietal beta power is opposite to other studies that showed increased power in occipital (45, 46) and temporal regions (52, 53). It is consistent, however, with an early report of beta power at parietal areas being involved in cognitive tasks (76). The analysis of the load effects therefore indicates that in patients there was substantially less load-dependent modulation of WM functions suggesting that independent of load, patients were closer to their WM capacity limits.

Healthy controls showed the expected anti-correlation of the DMN and dAN. This is in agreement with their attributed functional role of external versus internal orientation of attention, respectively (62, 63, 68). Furthermore, better performance in cognitive tasks, indexed by shorter and less variable reaction times, was linked to higher anti-correlations of the DMN and dAN leading to the discussion of anti-correlation between dAN and DMN being a possible marker for efficient cognitive processing (64, 65). This finding could be refined by relating WM performance in healthy subjects to higher anti-correlations of two hub regions of these networks, namely, the medial prefrontal cortex (mPFC) of the DMN and the dlPFC of the dAN at resting state (77). In addition, the significant prestimulus effects, namely, a reduction of the DMN and an increased activation of the dAN during the high-load condition imply that control subjects allocated their processing resources accordingly to task difficulty.

On the other hand, patient data gave evidence of a reduced anti-correlation of the DMN and dAN from prestimulus until the end of the WM retention at load 5, supporting the hypothesis of a deviant orientation of attention to internal versus external events. In particular, in patients, there was a reduced activation of the dAN during the prestimulus period and reduced suppression of the DMN during the retention period. Findings of altered activation of the dlPFC, a hub region of the dAN that is crucially involved in WM performance were frequently reported in schizophrenia. However, depending on the study, both under- as well as over-activations of the dlPFC have been discovered (12–16). As reported in the introduction, deviations in the form of reduced DMN suppression during WM tasks in schizophrenia are also well established and could be extended to youth at high-risk as well as unaffected siblings (31–34). Consequently, these results suggest that in patients there is a less coherent pattern of task-dependent preparatory processes.

Additionally, we found a missing lateralization effect regarding the recruitment of WMNs at higher load in patients. In controls, we observed a higher involvement of the left WMN over the whole trial duration. Patients in contrast showed less activation of the left WMN and a significantly higher involvement of right WMN during the retention period. In accordance with a study revealing relevance of left hemispheric WMN for verbal compared to right for spatial WM tasks in healthy subjects and its absence in patients (78), this finding could be interpreted either as inefficient inhibition of the task-irrelevant hemisphere or might be a compensation effect. The interpretation of this finding is hampered by controversial reports about hemispheric dominance in the literature. Although some studies seem to support left-sided dominance for verbal and right for spatial and object information (79), others showed bilateral involvement of the dlPFC as part of the WMNs (80) or even right hemispheric dominance, but left-sided being more commonly reported (81). These differences might be related to specifics of the WM tasks as well as methodologies of TCN extractions and definitions. It thus remains a question for future research to address.

Coming to the central aim of the present study, namely, the interaction of prestimulus TCN dynamics with task-related EEG spectral changes, the previous findings could be at least partly accounted for by the results from the covariance analyses. These covariance analyses can be considered as a biological fingerprint of the mechanisms that link preparatory activity captured by systematic prestimulus dynamics of specific TCNs to poststimulus, content-related processes.

First, in controls, the activation level of prestimulus DMN was negatively related to FM EEG theta during WM retention in a load-dependent manner. In patients, we found no FM effect for more difficult trials. Based on a previous finding that the stronger the inverse relationship between DMN and FM theta the better WM performance (82), we argue that a decreased effect in patients impacts their WM performance. In controls, the covariance map of the DMN with theta band at higher WM load resembled the one from the preceding study of Kottlow et al. (46). Therefore, we can argue that their map gave evidence that there are mechanisms through which prestimulus task dependent and therefore adaptive changes of TCN dynamics enhance task execution. The fact that the covariance map of the DMN was significantly different in patients and did not resemble a reversed EEG load effect indicates that such pre- to poststimulus processes did not affect the same poststimulus processes as in controls. This is especially interesting considering that the DMN activity did not differ significantly between groups in the prestimulus period, suggesting they were equally prepared for the task, but could not rally the necessary increase in theta band activity. During resting state, the study of Razavi et al. (55) found that for the DMN, the covariance maps of patients in the theta and alpha band were similar to the ones of controls in the beta band, indicating that the functional coupling of the DMN is changed in patients. Furthermore, as mentioned earlier there is the evidence of heightened theta power at rest in this patient population, which might partly explain the disclosed differences too. As our reported findings refer to task-related activations, it remains to be solved how the different coupling of the DMN with theta band can be explained. However, this finding demonstrates a difference in the functional state of the DMN in schizophrenia patients and might explain alterations in their cognitive processing.

Second, besides the reduced activation of patients’ dAN before encoding memoranda of five items, the coupling of the dAN with theta at lower load resembled the one at higher load in controls. This may be explained by the hypothesis that the retention of five items for controls and two items for patients depended on similar prestimulus attentional processes. The absence of such a link in controls at load 2 may then indicate that such attentional processes were irrelevant, whereas patients at load 5 were at their capacity limits and we observed a ceiling effect. The results of their performance showed the same pattern with the accuracy for patients at lower load (94.12%) being close to the one for controls at higher load (93.93%). The finding is in line with the left-shifted inverted *U*-shaped relationship between dlPFC activation and WM load in schizophrenia (74, 83, 84): higher activation levels are found in the dlPFC in patients at lower WM loads as well as reduced activity with increasing error rates at higher memory loads. Consequently, the WM system of patients seems to reach its capacity limits earlier (12, 13, 18, 74). This effect has been found not only in the dlPFC but also in the right parietal and left cingulate regions (83).

To conclude, the reported findings favor the view that in patients with schizophrenia is not only the balance of up- and downregulation of functional brain networks altered but also the relationship between pre-encoding activation and EEG power later during the retention interval. Despite only including trials with correct performance, we argue that the data presented here might at least partly explain well-known deficits in cognitive tasks such as WM. Furthermore, the findings of altered power spectra in patients during resting state indicates that these impairments might be of more generalized nature than only during a WM task as here investigated. Future studies should address the question of these impairments being rather state or trait markers. This work provides new insights regarding WM processing in schizophrenia and might motivate possible treatment strategies such as neurofeedback (85, 86) targeting preparatory brain states as for example the dAN, which showed a lack of anticipatory activation patterns in patients as an important factor for cognitive functioning in this disorder. Still, the specificity of these findings to WM performance needs to be proven.

One limitation of the study is the restriction of data due to the lack of both consistent load effects and covariance maps across groups. However, this is a meaningful outcome as patients showed less topographic consistencies both of relative EEG load effects and of their coupling of TCNs with EEG driving frequencies than healthy controls. The few consistent covariance maps for TCNs with beta band for both groups were in line with the finding of weaker load-dependent beta band effects, which might implicate that beta band was not crucially involved for successful WM performance. Somehow conflicting was the result of the right WMN coupled with alpha band at load 5 in patients being similar to load 2 in controls. The topography of the covariance map is comparable to previous studies at rest and during WM, but we might only speculate about the possible reason for the finding of coupling at higher load with alpha in patients resembling the one at lower load in controls. Therefore, we suggest that this finding should be taken cautiously and needs further evaluations. Other aspects for further investigations is the sensitivity of the time windows being crucial for successful WM performance, as we found different time windows to be critically different between controls and patients with schizophrenia, and the extension of the selection of TCN templates to be investigated. Finally, even though we took the medication dosage as covariate into account in our analyses, there is a study indicating that atypical medication other than clozapine, which has been excluded here, can lead to EEG abnormalities in patients and that these abnormalities are not correlated to the chlorpromazine equivalence dosage (87). However, the analyses of EEG correlates of TCN fluctuations and the analyses of the load effects are mathematically independent of constant changes in EEG spectral power. So, if one assumes that drug-induced EEG effects do not significantly interact with the experimental task, medication is not expected to play a significant role.

## Author Contributions

AB and TK wrote the manuscript; AB, LDH, and KR performed the measurements; AB, MK, and TK conceived and implemented experimental procedures and scripts for analyses; AB, TK, and JMF critically revised the manuscript.

## Conflict of Interest Statement

The authors declare that the research was conducted in the absence of any commercial or financial relationships that could be construed as a potential conflict of interest.

## Footnotes

^1^http://www.fil.ion.ucl.ac.uk/spm

^2^http://mialab.mrn.org/software/gift/

^3^http://www.thomaskoenig.ch/Ragu.htm

## References

[1] Baddeley A. Working memory. Science (1992) 255:556–9. 10.1126/science.1736359 1736359

[2] Weinberger DR Egan MF Bertolino A Callicott JH Mattay VS Lipska BK Prefrontal neurons and the genetics of schizophrenia. Biol Psychiatry (2001) 50:825–44. 10.1016/S0006-3223(01)01252-5 11743939

[3] Gold JM. Cognitive deficits as treatment targets in schizophrenia. Schizophr Res (2004) 72:21–8. 10.1016/j.schres.2004.09.008 15531404

[4] Green MF Cognitive impairment and functional outcome in schizophrenia and bipolar disorder. J Clin Psychiatry (2006) 67:e12 10.4088/JCP.1006e12 17107235

[5] Schaefer J Giangrande E Weinberger DR Dickinson D. The global cognitive impairment in schizophrenia: consistent over decades and around the world. Schizophr Res (2013) 150:42–50. 10.1016/j.schres.2013.07.009 23911259 PMC4196267

[6] Reichenberg A Harvey PD. Neuropsychological impairments in schizophrenia: integration of performance-based and brain imaging findings. Psychol Bull (2007) 133:833–58. 10.1037/0033-2909.133.5.833 17723032

[7] Hyde TM Nawroz S Goldberg TE Bigelow LB Strong D Ostrem JL Is there cognitive decline in schizophrenia? A cross-sectional study. Br J Psychiatry (1994) 164:494–500. 10.1192/bjp.164.4.494 8038938

[8] Hill SK Schuepbach D Herbener ES Keshavan MS Sweeney JA Pretreatment and longitudinal studies of neuropsychological deficits in antipsychotic-naive patients with schizophrenia. Schizophr Res (2004) 68:49–63. 10.1016/S0920-9964(03)00213-5 15037339

[9] Goldberg TE Weinberger DR. Effects of neuroleptic medications on the cognition of patients with schizophrenia: a review of recent studies. J Clin Psychiatry (1996) 57(Suppl 9):62–5. 8823353

[10] Couture SM Granholm EL Fish SC. A path model investigation of neurocognition, theory of mind, social competence, negative symptoms and real-world functioning in schizophrenia. Schizophr Res (2011) 125:152–60. 10.1016/j.schres.2010.09.020 20965699 PMC3031755

[11] Mancuso F Horan WP Kern RS Green MF. Social cognition in psychosis: multidimensional structure, clinical correlates, and relationship with functional outcome. Schizophr Res (2011) 125:143–51. 10.1016/j.schres.2010.11.007 21112743 PMC3073542

[12] Manoach DS Press DZ Thangaraj V Searl MM Goff DC Halpern E Schizophrenic subjects activate dorsolateral prefrontal cortex during a working memory task, as measured by fMRI. Biol Psychiatry (1999) 45:1128–37. 10.1016/S0006-3223(98)00318-7 10331104

[13] Manoach DS Gollub RL Benson ES Searl MM Goff DC Halpern E Schizophrenic subjects show aberrant fMRI activation of dorsolateral prefrontal cortex and basal ganglia during working memory performance. Biol Psychiatry (2000) 48:99–109. 10.1016/S0006-3223(00)00227-4 10903406

[14] Johnson MR Morris NA Astur RS Calhoun VD Mathalon DH Kiehl KA A functional magnetic resonance imaging study of working memory abnormalities in schizophrenia. Biol Psychiatry (2006) 60:11–21. 10.1016/j.biopsych.2005.11.012 16503328

[15] Potkin SG Turner JA Brown GG McCarthy G Greve DN Glover GH Working memory and DLPFC inefficiency in schizophrenia: the FBIRN study. Schizophr Bull (2009) 35:19–31. 10.1093/schbul/sbn162 19042912 PMC2643959

[16] Quide Y Morris RW Shepherd AM Rowland JE Green MJ. Task-related fronto-striatal functional connectivity during working memory performance in schizophrenia. Schizophr Res (2013) 150:468–75. 10.1016/j.schres.2013.08.009 24016726

[17] Glahn DC Ragland JD Abramoff A Barrett J Laird AR Bearden CE Beyond hypofrontality: a quantitative meta-analysis of functional neuroimaging studies of working memory in schizophrenia. Hum Brain Mapp (2005) 25:60–9. 10.1002/hbm.20138 15846819 PMC6871703

[18] Metzak PD Riley JD Wang L Whitman JC Ngan ET Woodward TS. Decreased efficiency of task-positive and task-negative networks during working memory in schizophrenia. Schizophr Bull (2012) 38:803–13. 10.1093/schbul/sbq154 21224491 PMC3406536

[19] Brandt CL Eichele T Melle I Sundet K Server A Agartz I Working memory networks and activation patterns in schizophrenia and bipolar disorder: comparison with healthy controls. Br J Psychiatry (2014) 204:290–8. 10.1192/bjp.bp.113.129254 24434074

[20] Calhoun VD Kiehl KA Pearlson GD. Modulation of temporally coherent brain networks estimated using ICA at rest and during cognitive tasks. Hum Brain Mapp (2008) 29:828–38. 10.1002/hbm.20581 18438867 PMC2649823

[21] Liang M Zhou Y Jiang T Liu Z Tian L Liu H Widespread functional disconnectivity in schizophrenia with resting-state functional magnetic resonance imaging. Neuroreport (2006) 17:209–13. 10.1097/01.wnr.0000198434.06518.b8 16407773

[22] Bluhm RL Miller J Lanius RA Osuch EA Boksman K Neufeld RW Spontaneous low-frequency fluctuations in the BOLD signal in schizophrenic patients: anomalies in the default network. Schizophr Bull (2007) 33:1004–12. 10.1093/schbul/sbm052 17556752 PMC2632312

[23] Zhou Y Liang M Tian L Wang K Hao Y Liu H Functional disintegration in paranoid schizophrenia using resting-state fMRI. Schizophr Res (2007) 97:194–205. 10.1016/j.schres.2007.05.029 17628434

[24] Jafri MJ Pearlson GD Stevens M Calhoun VD. A method for functional network connectivity among spatially independent resting-state components in schizophrenia. Neuroimage (2008) 39:1666–81. 10.1016/j.neuroimage.2007.11.001 18082428 PMC3164840

[25] Littow H Huossa V Karjalainen S Jaaskelainen E Haapea M Miettunen J Aberrant functional connectivity in the default mode and central executive networks in subjects with schizophrenia – a whole-brain resting-state ICA study. Front Psychiatry (2015) 6:26. 10.3389/fpsyt.2015.00026 25767449 PMC4341512

[26] Garrity AG Pearlson GD McKiernan K Lloyd D Kiehl KA Calhoun VD. Aberrant “default mode” functional connectivity in schizophrenia. Am J Psychiatry (2007) 164:450–7. 10.1176/appi.ajp.164.3.450 17329470

[27] Pomarol-Clotet E Salvador R Sarro S Gomar J Vila F Martinez A Failure to deactivate in the prefrontal cortex in schizophrenia: dysfunction of the default mode network? Psychol Med (2008) 38:1185–93. 10.1017/S0033291708003565 18507885

[28] McKiernan KA Kaufman JN Kucera-Thompson J Binder JR. A parametric manipulation of factors affecting task-induced deactivation in functional neuroimaging. J Cogn Neurosci (2003) 15:394–408. 10.1162/089892903321593117 12729491

[29] Daselaar SM Prince SE Cabeza R. When less means more: deactivations during encoding that predict subsequent memory. Neuroimage (2004) 23:921–7. 10.1016/j.neuroimage.2004.07.031 15528092

[30] Anticevic A Repovs G Shulman GL Barch DM. When less is more: TPJ and default network deactivation during encoding predicts working memory performance. Neuroimage (2010) 49:2638–48. 10.1016/j.neuroimage.2009.11.008 19913622 PMC3226712

[31] Kim DI Manoach DS Mathalon DH Turner JA Mannell M Brown GG Dysregulation of working memory and default-mode networks in schizophrenia using independent component analysis, an fBIRN and MCIC study. Hum Brain Mapp (2009) 30:3795–811. 10.1002/hbm.20807 19434601 PMC3058491

[32] Whitfield-Gabrieli S Thermenos HW Milanovic S Tsuang MT Faraone SV McCarley RW Hyperactivity and hyperconnectivity of the default network in schizophrenia and in first-degree relatives of persons with schizophrenia. Proc Natl Acad Sci U S A (2009) 106:1279–84. 10.1073/pnas.0809141106 19164577 PMC2633557

[33] Fryer SL Woods SW Kiehl KA Calhoun VD Pearlson GD Roach BJ Deficient suppression of default mode regions during working memory in individuals with early psychosis and at clinical high-risk for psychosis. Front Psychiatry (2013) 4:92. 10.3389/fpsyt.2013.00092 24032017 PMC3768116

[34] de Leeuw M Kahn RS Zandbelt BB Widschwendter CG Vink M. Working memory and default mode network abnormalities in unaffected siblings of schizophrenia patients. Schizophr Res (2013) 150:555–62. 10.1016/j.schres.2013.08.016 24051015

[35] Koukkou M Lehmann D. Dreaming: the functional state-shift hypothesis. A neuropsychophysiological model. Br J Psychiatry (1983) 142:221–31. 10.1192/bjp.142.3.221 6860875

[36] Eichele T Debener S Calhoun VD Specht K Engel AK Hugdahl K Prediction of human errors by maladaptive changes in event-related brain networks. Proc Natl Acad Sci U S A (2008) 105:6173–8. 10.1073/pnas.0708965105 18427123 PMC2329680

[37] Li CS Yan P Bergquist KL Sinha R. Greater activation of the “default” brain regions predicts stop signal errors. Neuroimage (2007) 38:640–8. 10.1016/j.neuroimage.2007.07.021 17884586 PMC2097963

[38] Ives JR Warach S Schmitt F Edelman RR Schomer DL. Monitoring the patient’s EEG during echo planar MRI. Electroencephalogr Clin Neurophysiol (1993) 87:417–20. 10.1016/0013-4694(93)91206-G 7508375

[39] Vitali P Di Perri C Vaudano AE Meletti S Villani F. Integration of multimodal neuroimaging methods: a rationale for clinical applications of simultaneous EEG-fMRI. Funct Neurol (2015) 30:9–20. 10.11138/FNeur/2015.30.1.009 26214023 PMC4520679

[40] Scheeringa R Bastiaansen MC Petersson KM Oostenveld R Norris DG Hagoort P. Frontal theta EEG activity correlates negatively with the default mode network in resting state. Int J Psychophysiol (2008) 67:242–51. 10.1016/j.ijpsycho.2007.05.017 17707538

[41] Gevins A Smith ME McEvoy L Yu D. High-resolution EEG mapping of cortical activation related to working memory: effects of task difficulty, type of processing, and practice. Cereb Cortex (1997) 7:374–85. 10.1093/cercor/7.4.374 9177767

[42] Jensen O Tesche CD. Frontal theta activity in humans increases with memory load in a working memory task. Eur J Neurosci (2002) 15:1395–9. 10.1046/j.1460-9568.2002.01975.x 11994134

[43] Onton J Delorme A Makeig S. Frontal midline EEG dynamics during working memory. Neuroimage (2005) 27:341–56. 10.1016/j.neuroimage.2005.04.014 15927487

[44] Khader PH Jost K Ranganath C Rosler F. Theta and alpha oscillations during working-memory maintenance predict successful long-term memory encoding. Neurosci Lett (2010) 468:339–43. 10.1016/j.neulet.2009.11.028 19922772 PMC3951969

[45] Michels L Bucher K Luchinger R Klaver P Martin E Jeanmonod D Simultaneous EEG-fMRI during a working memory task: modulations in low and high frequency bands. PLoS One (2010) 5:e10298. 10.1371/journal.pone.0010298 20421978 PMC2858659

[46] Kottlow M Schlaepfer A Baenninger A Michels L Brandeis D Koenig T. Pre-stimulus BOLD-network activation modulates EEG spectral activity during working memory retention. Front Behav Neurosci (2015) 9:111. 10.3389/fnbeh.2015.00111 25999828 PMC4422031

[47] Schmiedt C Brand A Hildebrandt H Basar-Eroglu C. Event-related theta oscillations during working memory tasks in patients with schizophrenia and healthy controls. Brain Res Cogn Brain Res (2005) 25:936–47. 10.1016/j.cogbrainres.2005.09.015 16289526

[48] Haenschel C Bittner RA Waltz J Haertling F Wibral M Singer W Cortical oscillatory activity is critical for working memory as revealed by deficits in early-onset schizophrenia. J Neurosci (2009) 29:9481–9. 10.1523/JNEUROSCI.1428-09.2009 19641111 PMC6666530

[49] Jensen O Gelfand J Kounios J Lisman JE. Oscillations in the alpha band (9-12 Hz) increase with memory load during retention in a short-term memory task. Cereb Cortex (2002) 12:877–82. 10.1093/cercor/12.8.877 12122036

[50] Schack B Klimesch W. Frequency characteristics of evoked and oscillatory electroencephalic activity in a human memory scanning task. Neurosci Lett (2002) 331:107–10. 10.1016/S0304-3940(02)00846-7 12361852

[51] Michels L Moazami-Goudarzi M Jeanmonod D Sarnthein J. EEG alpha distinguishes between cuneal and precuneal activation in working memory. Neuroimage (2008) 40:1296–310. 10.1016/j.neuroimage.2007.12.048 18272404

[52] Leiberg S Lutzenberger W Kaiser J. Effects of memory load on cortical oscillatory activity during auditory pattern working memory. Brain Res (2006) 1120:131–40. 10.1016/j.brainres.2006.08.066 16989782

[53] Stokic M Milovanovic D Ljubisavljevic MR Nenadovic V Cukic M. Memory load effect in auditory-verbal short-term memory task: EEG fractal and spectral analysis. Exp Brain Res (2015) 233(10):3023–38. 10.1007/s00221-015-4372-z 26169106

[54] Jann K Kottlow M Dierks T Boesch C Koenig T. Topographic electrophysiological signatures of fMRI resting state networks. PLoS One (2010) 5:e12945. 10.1371/journal.pone.0012945 20877577 PMC2943931

[55] Razavi N Jann K Koenig T Kottlow M Hauf M Strik W Shifted coupling of EEG driving frequencies and fMRI resting state networks in schizophrenia spectrum disorders. PLoS One (2013) 8:e76604. 10.1371/journal.pone.0076604 24124576 PMC3790692

[56] Boutros NN Arfken C Galderisi S Warrick J Pratt G Iacono W. The status of spectral EEG abnormality as a diagnostic test for schizophrenia. Schizophr Res (2008) 99:225–37. 10.1016/j.schres.2007.11.020 18160260 PMC2288752

[57] Treves IA Neufeld MY. EEG abnormalities in clozapine-treated schizophrenic patients. Eur Neuropsychopharmacol (1996) 6:93–4. 10.1016/0924-977X(95)00057-V 8791033

[58] Sternberg S. High-speed scanning in human memory. Science (1966) 153:652–4. 10.1126/science.153.3736.652 5939936

[59] Kottlow M Jann K Dierks T Koenig T. Increased phase synchronization during continuous face integration measured simultaneously with EEG and fMRI. Clin Neurophysiol (2012) 123:1536–48. 10.1016/j.clinph.2011.12.019 22305306

[60] Allen PJ Josephs O Turner R. A method for removing imaging artifact from continuous EEG recorded during functional MRI. Neuroimage (2000) 12:230–9. 10.1006/nimg.2000.0599 10913328

[61] Calhoun VD Adali T Pearlson GD Pekar JJ. A method for making group inferences from functional MRI data using independent component analysis. Hum Brain Mapp (2001) 14:140–51. 10.1002/hbm.1048 11559959 PMC6871952

[62] Fox MD Snyder AZ Vincent JL Corbetta M Van Essen DC Raichle ME. The human brain is intrinsically organized into dynamic, anticorrelated functional networks. Proc Natl Acad Sci U S A (2005) 102:9673–8. 10.1073/pnas.0504136102 15976020 PMC1157105

[63] Fransson P. Spontaneous low-frequency BOLD signal fluctuations: an fMRI investigation of the resting-state default mode of brain function hypothesis. Hum Brain Mapp (2005) 26:15–29. 10.1002/hbm.20113 15852468 PMC6871700

[64] Kelly AM Uddin LQ Biswal BB Castellanos FX Milham MP. Competition between functional brain networks mediates behavioral variability. Neuroimage (2008) 39:527–37. 10.1016/j.neuroimage.2007.08.008 17919929

[65] De Pisapia N Turatto M Lin P Jovicich J Caramazza A. Unconscious priming instructions modulate activity in default and executive networks of the human brain. Cereb Cortex (2012) 22:639–49. 10.1093/cercor/bhr146 21690258

[66] Koenig T Kottlow M Stein M Melie-Garcia L. Ragu: a free tool for the analysis of EEG and MEG event-related scalp field data using global randomization statistics. Comput Intell Neurosci (2011) 2011:938925. 10.1155/2011/938925 21403863 PMC3049349

[67] Koenig T Melie-Garcia L Stein M Strik W Lehmann C. Establishing correlations of scalp field maps with other experimental variables using covariance analysis and resampling methods. Clin Neurophysiol (2008) 119:1262–70. 10.1016/j.clinph.2007.12.023 18424230

[68] Kim H. Encoding and retrieval along the long axis of the hippocampus and their relationships with dorsal attention and default mode networks: the HERNET model. Hippocampus (2015) 25:500–10. 10.1002/hipo.22387 25367784

[69] Itil TM Qualitative and quantitative EEG-findings in schizophrenia. EEG EMG Z Elektroenzephalogr Elektromyogr Verwandte Geb (1978) 9:1–13. 416942

[70] Galderisi S Mucci A Mignone ML Maj M Kemali D. CEEG mapping in drug-free schizophrenics. Differences from healthy subjects and changes induced by haloperidol treatment. Schizophr Res (1991) 6:15–23. 10.1016/0920-9964(91)90016-K 1786232

[71] Sponheim SR Clementz BA Iacono WG Beiser M. Clinical and biological concomitants of resting state EEG power abnormalities in schizophrenia. Biol Psychiatry (2000) 48:1088–97. 10.1016/S0006-3223(00)00907-0 11094142

[72] Galderisi S Mucci A Volpe U Boutros N. Evidence-based medicine and electrophysiology in schizophrenia. Clin EEG Neurosci (2009) 40:62–77. 10.1177/155005940904000206 19534300

[73] Klein C Diaz Hernandez L Koenig T Kottlow M Elmer S Jancke L. The influence of pre-stimulus EEG activity on reaction time during a verbal sternberg task is related to musical expertise. Brain Topogr (2016) 29:67–81. 10.1007/s10548-015-0433-7 25929715

[74] Cairo TA Woodward TS Ngan ET. Decreased encoding efficiency in schizophrenia. Biol Psychiatry (2006) 59:740–6. 10.1016/j.biopsych.2005.08.009 16229823

[75] Nenert R Viswanathan S Dubuc DM Visscher KM. Modulations of ongoing alpha oscillations predict successful short-term visual memory encoding. Front Hum Neurosci (2012) 6:127. 10.3389/fnhum.2012.00127 22586390 PMC3347628

[76] Ray WJ Cole HW. EEG alpha activity reflects attentional demands, and beta activity reflects emotional and cognitive processes. Science (1985) 228:750–2. 10.1126/science.3992243 3992243

[77] Keller JB Hedden T Thompson TW Anteraper SA Gabrieli JD Whitfield-Gabrieli S. Resting-state anticorrelations between medial and lateral prefrontal cortex: association with working memory, aging, and individual differences. Cortex (2015) 64:271–80. 10.1016/j.cortex.2014.12.001 25562175 PMC4346444

[78] Walter H Wunderlich AP Blankenhorn M Schafer S Tomczak R Spitzer M No hypofrontality, but absence of prefrontal lateralization comparing verbal and spatial working memory in schizophrenia. Schizophr Res (2003) 61:175–84. 10.1016/S0920-9964(02)00225-6 12729869

[79] Fletcher PC Henson RN. Frontal lobes and human memory: insights from functional neuroimaging. Brain (2001) 124:849–81. 10.1093/brain/124.5.849 11335690

[80] Wager TD Smith EE. Neuroimaging studies of working memory: a meta-analysis. Cogn Affect Behav Neurosci (2003) 3:255–74. 10.3758/CABN.3.4.255 15040547

[81] Nee DE Brown JW Askren MK Berman MG Demiralp E Krawitz A A meta-analysis of executive components of working memory. Cereb Cortex (2013) 23:264–82. 10.1093/cercor/bhs007 22314046 PMC3584956

[82] White TP Jansen M Doege K Mullinger KJ Park SB Liddle EB Theta power during encoding predicts subsequent-memory performance and default mode network deactivation. Hum Brain Mapp (2013) 34:2929–43. 10.1002/hbm.22114 22711646 PMC6870261

[83] Callicott JH Bertolino A Mattay VS Langheim FJ Duyn J Coppola R Physiological dysfunction of the dorsolateral prefrontal cortex in schizophrenia revisited. Cereb Cortex (2000) 10:1078–92. 10.1093/cercor/10.11.1078 11053229

[84] Callicott JH Mattay VS Verchinski BA Marenco S Egan MF Weinberger DR. Complexity of prefrontal cortical dysfunction in schizophrenia: more than up or down. Am J Psychiatry (2003) 160:2209–15. 10.1176/appi.ajp.160.12.2209 14638592

[85] Diaz Hernandez L Rieger K Baenninger A Brandeis D Koenig T Towards using microstate-neurofeedback for the treatment of psychotic symptoms in schizophrenia. A feasibility study in healthy participants. Brain Topogr (2015) 29(2):308–21. 10.1007/s10548-015-0460-4 26582260

[86] Zhang Q Zhang G Yao L Zhao X. Impact of real-time fMRI working memory feedback training on the interactions between three core brain networks. Front Behav Neurosci (2015) 9:244. 10.3389/fnbeh.2015.00244 26388754 PMC4559651

[87] Centorrino F Price BH Tuttle M Bahk WM Hennen J Albert MJ EEG abnormalities during treatment with typical and atypical antipsychotics. Am J Psychiatry (2002) 159:109–15. 10.1176/appi.ajp.159.1.109 11772698

