# Corrigendum: Inefficient Preparatory fMRI-BOLD Network Activations Predict Working Memory Dysfunctions in Patients with Schizophrenia

**DOI:** 10.3389/fpsyt.2016.00141

**Published:** 2016-08-29

**Authors:** Anja Baenninger, Laura Diaz Hernandez, Kathryn Rieger, Judith M. Ford, Mara Kottlow, Thomas Koenig

**Affiliations:** ^1^Translational Research Center, University Hospital of Psychiatry and Psychotherapy, University of Bern, Bern, Switzerland; ^2^San Francisco VA Medical Center, San Francisco, CA, USA; ^3^Center for Cognition, Learning and Memory, University of Bern, Bern, Switzerland; ^4^Department of Psychiatry, University of California San Francisco, San Francisco, CA, USA

**Keywords:** schizophrenia, working memory, temporally coherent networks, state-dependent information processing, simultaneous EEG-fMRI, covariance mapping

**Reason for Corrigendum:**

There is a mistake in the labeling of the two networks plotted in Figure 4: the default mode network (DMN) of controls should be indicated with circles instead of triangles such as the dorsal attention network (dAN). The authors apologize for the mistake. The error does not change the content of the article in any way.

In Figure 5, there was mistakenly a red correction line below the label of the right working memory network (rWMN) that was unfortunately not spotted upon publication. This change has no impact neither on the scientific work nor the conclusions of the article in any way. We apologize for the oversight.

The corrected Figures are below:

**Figure 4:**
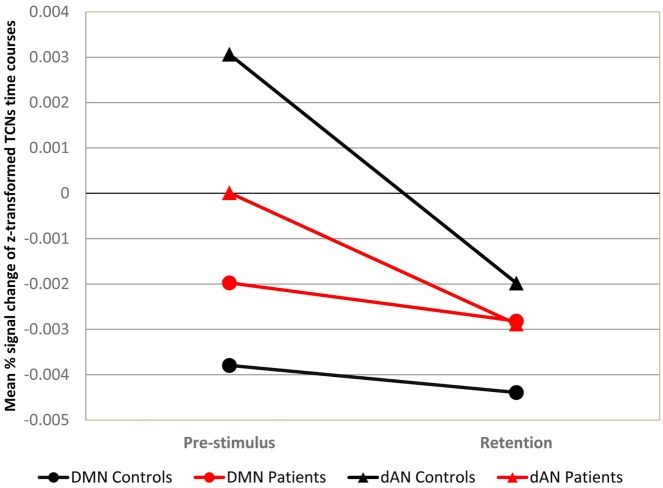
**Mean DMN and dAN from pre- to poststimulus in the WM task**. Mean DMN and dAN dynamics at prestimulus and retention intervals for each load and group. *X*-axis: time points (prestimulus and retention period), *Y*-axis: mean percent signal change of variance normalized, and z-transformed TCNs.

**Figure 5:**
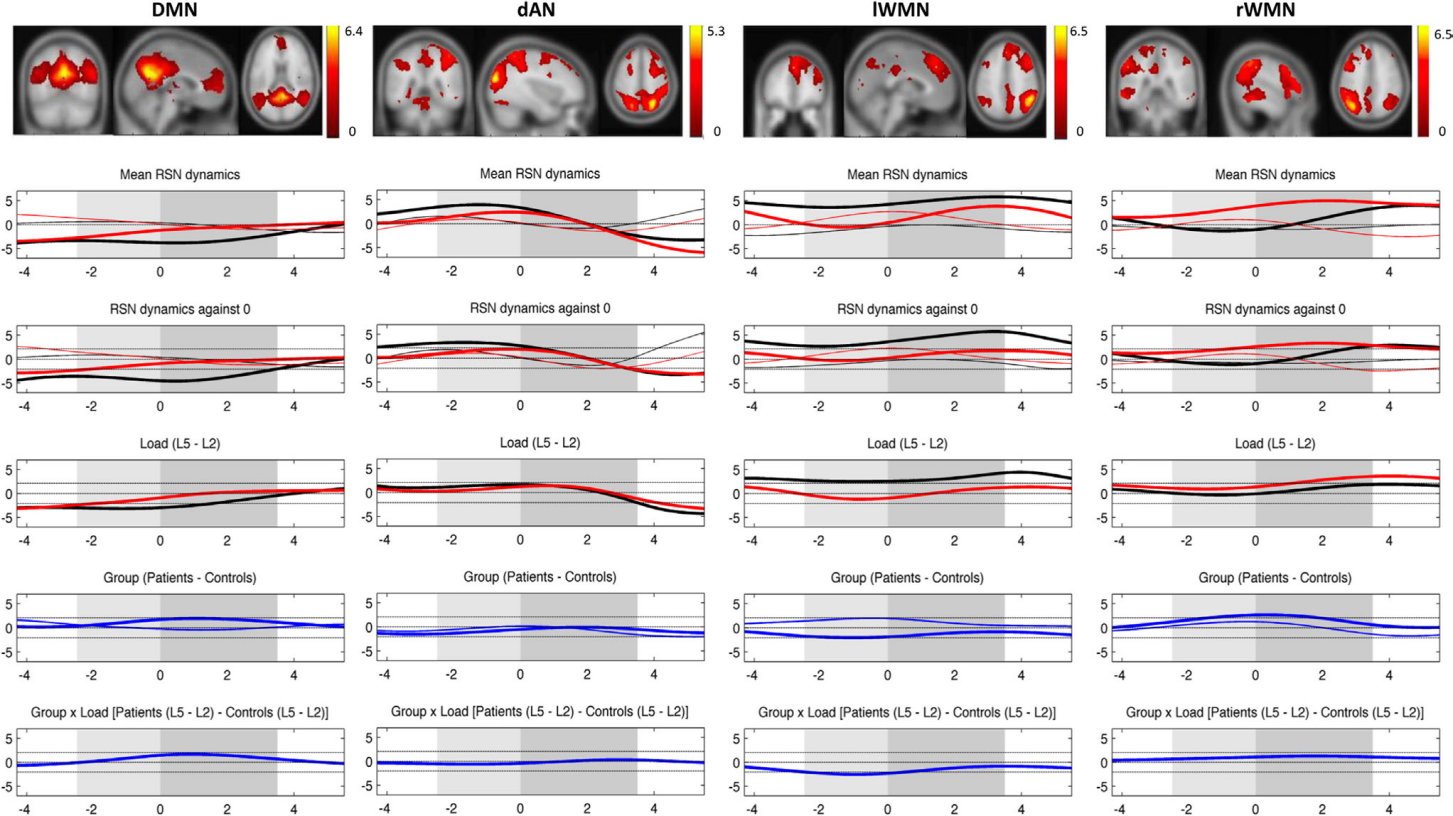
**Mean evolution of TCNs over average trials**. Upper plot shows the templates for each TCN from the study of Kottlow et al. with the three orthogonal slices through areas of maximum activation (only positive values; for detailed information about included regions, see Table S1 in Supplementary Material). Lower plots display the mean network evolutions over average trials for each load (thin line = load 2; thick line = load 5) and each group (black line = controls; red line = patients). Dashed lines indicate significance of the *t*-tests (two-sided, *p* = 0.05): (1) mean evolutions, (2) mean evolutions against 0, (3) load effect, (4) group effect (blue thin line = load 2; blue thick line = load 5), and (5) interaction of group by load. *X*-axis: time over trial [prestimulus: −4 to −2.5 s; stimulus (light gray block): −2.5 to 0 s; retention (dark gray block): 0–3.5 s; probe: 3.5–5.5 s], *Y*-axis: percent signal change of variance normalized, and *z*-transformed TCNs’ time courses.

## Conflict of Interest Statement

The authors declare that the research was conducted in the absence of any commercial or financial relationships that could be construed as a potential conflict of interest.

