# Supplementary material for: Inefficient Preparatory fMRI-BOLD Network Activations Predict Working Memory Dysfunctions in Patients with Schizophrenia
Source: Front Psychiatry. 2016 Mar 18;7:29. doi: 10.3389/fpsyt.2016.00029 (PMC4796005; doi:10.3389/fpsyt.2016.00029)

Supplementary Material

Inefficient preparatory fMRI-BOLD network activations predict working memory dysfunctions in patients with schizophrenia

Anja Baenninger*, Laura Diaz Hernandez, Kathryn Rieger, Judith M. Ford, Mara Kottlow, Thomas Koenig

*** Correspondence:** Anja Baenninger: anja.baenninger@puk.unibe.ch

1.1 Supplementary Tables

Supplementary Table 1. Regions of the four template TCNs from Kottlow et al. Using the "write Talairach table" option based on the Talairach Daemon of the gift toolbox, max values and labeling of the coordinates were extracted. A threshold of 3.5 was applied for beta-values and the distance between contiguous voxels was set to 4mm. Only positive regions are listed.

TCN	Region	Random effects: Max Value (x,y,z)
**DMN**	Precuneus	9.2 (-20, -66, 49) / 11.6 (30, -72, 35)
	Superior Parietal Lobule	9.4 (-24, -55, 60) / 11.4 (22, -59, 56)
	Middle Temporal Gyrus	8.6 (-34, -76, 24) / 11.4 (38, -76, 24)
	Superior Occipital Gyrus	8.9 (-32, -78, 28) / 10.8 (38, -76, 28)
	Angular Gyrus	6.3 (-34, -74, 31) / 10.0 (36, -72, 31)
	Middle Occipital Gyrus	8.9 (-32, -82, 21) / 9.3 (34, -81, 21)
	Inferior Parietal Lobule	6.4 (-34, -50, 56) / 8.7 (44, -36, 53)
	Postcentral Gyrus	5.9 (-18, -51, 63) / 8.2 (44, -34, 50)
	Cuneus	7.6 (-28, -80, 32) / 7.5 (30, -80, 32)

	Middle Frontal Gyrus	4.6 (-26, 3, 55) / 6.7 (30, 9, 60)
	Inferior Temporal Gyrus	6.5 (-53, -64, -2) / 5.1 (48, -70, -2)
	Superior Frontal Gyrus	5.0 (-24, 15, 58) / 6.4 (26, 7, 55)
	Inferior Frontal Gyrus	4.1 (-53, 13, 21) / 5.6 (48, 9, 22)
	Paracentral Lobule	3.7 (0, -38, 50) / 5.4 (4, -46, 59)
	Superior Temporal Gyrus	3.9 (-46, 6, -5) / 4.7 (48, -61, 18)
	Fusiform Gyrus	4.3 (-44, -67, -12) / 4.5 (48, -57, -12)
	Precentral Gyrus	4.5 (-55, -4, 39) / 4.0 (59, -17, 40)
	Culmen	4.4 (-16, -37, -12)/3.8 (2, -45, -8)
	Insula	4.2 (-42, 4, -4)
	Cingulate Gyrus	4.0 (6, -37, 41)
	Medial Frontal Gyrus	3.9 (-20, 3, 51)
	Inferior Occipital Gyrus	3.9 (-42, -70, -3)
	Thalamus	3.9 (16, -29, 12)
**dAN**	Precuneus	8.5 (-20, -66, 49) / 11.0 (30, -72, 35)
	Middle Temporal Gyrus	8.4 (-30, -75, 20) / 10.7 (38, -77, 22)
	Superior Occipital Gyrus	8.0 (-28, -80, 28) / 10.4 (38, -76, 26)
	Superior Parietal Lobule	8.6 (-24, -57, 58) / 10.2 (24, -57, 58)
	Angular Gyrus	5.9 (-34, -76, 31) / 9.6 (38, -76, 30)
	Middle Occitipital Gyrus	8.2 (-32, -79, 21) / 7.8 (36, -83, 19)

	Cuneus	8.0 (-26, -76, 31) / 7.9 (32, -80, 33)
	Inferior Parietal Lobule	6.7 (-30, -50, 54) / 7.9 (34, -50, 56)
	Postcentral Gyrus	4.5 (-6, -53, 65) / 7.3 (44, -34, 50)
	Middle Frontal Gyrus	5.5 (26, 0, 48)
	Inferior Frontal Gyrus	5.2 (48, 9, 24)
	Superior Frontal Gyrus	5.1 (26, 7, 55)
	Inferior Temporal Gyrus	4.5 (-53, -58, -4) / 4.9 (51, -61, -9)
	Precentral Gyrus	3.6 (-53, -2, 41) / 4.2 (59, -17, 41)
	Fusiform Gyrus	4.1 (50, -61, -12)
	Superior Temporal Gyrus	3.9 (48, -61, 18)
**rWMN**	Inferior Parietal Lobule	6.3 (-48, -52, 43) / 16.6 (46, -54, 47)
	Superior Parietal Lobule	4.3 (-38, -56, 51) / 16.4 (42, -58, 49)
	Supramarginal Gyrus	4.4 (-46, -51, 36) / 14.7 (51, -49, 37)
	Middle Frontal Gyrus	4.9 (-44, 50, -1) / 13.3 (46, 21, 39)
	Angular Gyrus	3.6 (-48, -55, 36) / 12.6 (48, -55, 36)
	Precentral Gyrus	11.9 (48, 21, 36)
	Superior Frontal Gyrus	6.1 (-2, 33, 48) / 11.7 (36, 22, 49)
	Medial Frontal Gyrus	7.1 (-2, 35, 42) / 10.2 (4, 35, 39)
	Middle Temporal Gyrus	4.3 (-63, -27, -5) / 9.9 (63, -26, -7)
	Postcentral Gyrus	9.3 (55, -36, 50)
	Inferior Frontal Gyrus	3.8 (-48, 47, 0) / 9.3 (40, 54, 1)

	Precuneus	3.6 (0, -70, 46) / 8.9 (36, -66, 42)
	Superior Temporal Gyrus	7.9 (48, -48, 21)
	Cingulate Gyrus	3.6 (-2, -26, 31) / 6.9 (10, -45, 37)
	Declive	6.6 (-10, -79, -20) / -999.0 (0, 0, 0)
	Anterior Cingulate	5.6 (8, 41, 13)
	Inferior Temporal Gyrus	4.8 (59, -14, -16)
	Insula	4.1 (48, -40, 20)
	Uvula	3.9 (-14, -71, -23)
	Cuneus	3.7 (10, -66, 31)
**lWMN**	Inferior Parietal Lobule	14.9 (-38, -62, 44) / 7.1 (34, -58, 40)
	Superior Parietal Lobule	14.6 (-36, -62, 49) / 6.3 (40, -58, 51)
	Precuneus	13.0 (-34, -64, 40) / 5.9 (32, -62, 40)
	Inferior Frontal Gyrus	11.5 (-46, 43, 5) / 5.5 (53, 38, 13)
	Angular Gyrus	11.1 (-46, -56, 36) / 5.4 (34, -58, 36)
	Supramarginal Gyrus	10.7 (-46, -53, 36) / 5.1 (38, -49, 37)
	Middle Frontal Gyrus	10.6 (-46, 44, -4) / 5.3 (50, 36, 18)
	Middle Temporal Gyrus	9.8 (-61, -37, -5) / 5.1 (65, -35, -5)
	Superior Temporal Gyrus	9.1 (-46, -57, 29) / 3.9 (63, -2, 7)
	Precentral Gyrus	8.0 (-44, 17, 34) / 3.5 (61, -1, 11)
	Superior Frontal Gyrus	7.6 (-34, 14, 51)

Inferior Temporal Gyrus	7.1 (-51, -53, -11)
Medial Frontal Gyrus	6.6 (-4, 29, 41)
Fusiform Gyrus	6.4 (-48, -55, -11)
Uvula	5.3 (32, -63, -24)
Cingulate Gyrus	5.0 (-2, -33, 33) / 3.8 (2, -33, 33)
Postcentral Gyrus	4.7 (-51, -31, 49) / 4.2 (53, -34, 51)
Superior Occipital Gyrus	4.5 (-38, -74, 28)
Culmen	4.2 (28, -61, -24)
Cuneus	4.0 (0, -90, 17)
Insula	3.6 (-40, 17, -1)

Supplementary Figure 1: Difference of spectral power in three frequency bands (theta, alpha, beta) comparing patients with controls during the resting state condition with eyes closed. Indicated with the dashed black line is the critical threshold for the significant t-value (2.0369; df = 32, *p* = .05, double-sided).

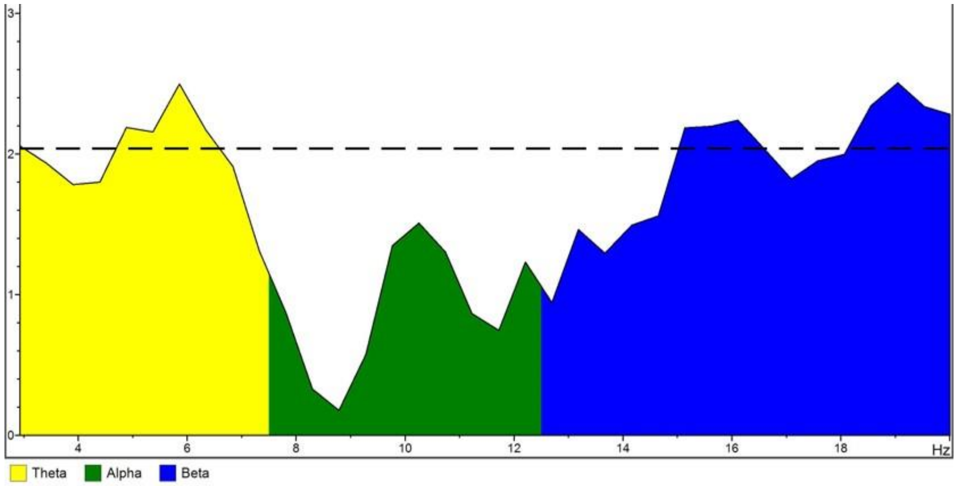

Supplement: Supplementary file 1 [file Data_Sheet_1.PDF]
